# Deduction of the operable design space of RP-HPLC technique for the simultaneous estimation of metformin, pioglitazone, and glimepiride

**DOI:** 10.1038/s41598-023-30051-x

**Published:** 2023-03-16

**Authors:** Aya A. Marie, Sherin F. Hammad, Mohamed M. Salim, Mahmoud M. Elkhodary, Amira H. Kamal

**Affiliations:** 1 Department of Pharmaceutical Analytical Chemistry, Faculty of Pharmacy, Tanta University, Tanta, 31527 Egypt; 2 Department of Pharmaceutical Chemistry, Faculty of Pharmacy, Horus University-Egypt, New Damietta, 34517 Egypt; 3 Department of Pharmaceutical Analytical Chemistry, Faculty of Pharmacy, Mansoura University, Mansoura, 35516 Egypt; 4 Faculty of Pharmacy, Medical Campus of Tanta University, Elgeish Street, Tanta, 31111 Egypt

**Keywords:** Analytical chemistry, Drug development

## Abstract

A reversed-phase RP-HPLC method was developed for the simultaneous determination of metformin hydrochloride (MET), pioglitazone (PIO), and glimepiride (GLM) in their combined dosage forms and spiked human plasma. Quality risk management principles for determining the critical method parameters (CMPs) and fractional factorial design were made to screen CMPs and subsequently, the Box–Behnken design was employed. The analytical Quality by Design (AQbD) paradigm was used to establish the method operable design region (MODR) for the developed method depended on understanding the quality target product profile (QTPP), analytical target profile (ATP), and risk assessment for different factors that affect the method performance to develop an accurate, precise, cost-effective, and environmentally benign method. The separation was carried out using a mobile phase composed of methanol: 0.05 M potassium dihydrogen phosphate buffer pH 3.7 with 0.05% TEA (78:22, v/v). The flow rate was 1.2 mL/min. DAD detector was set at 227 nm. Linagliptin (LIN) was used as an internal standard. The proposed method was validated according to The International Council for Harmonization of Technical Requirements for Pharmaceuticals for Human Use (ICH). The assay results obtained by using the developed method were statistically compared to those obtained by the reported HPLC method, and a satisfying agreement was observed.

## Introduction

Diabetes is one of the rapidly spreading health problems in Egypt with a substantial impact on morbidity, mortality, and health care problem^1^. The International Diabetes Federation (IDF) marks Egypt as The ninth-highest country in the world containing many diabetics.

Metformin (MET, Fig. S1a) is a biguanide, an oral antidiabetic drug^2^ for treating type-II diabetes^3^. It reduces glucose production from the liver and minimizes triglyceride and cholesterol levels^3^.

Pioglitazone (PIO, Fig. S1b) is a thiazolidinedione-type, also called "glitazones"^4^. Thiazolidinediones are Peroxisome Proliferator-Activated Receptor (PPAR-gamma) agonists, used for the treatment of diabetes type II. Pioglitazone is popular to be active in controlling glycemic by reducing insulin resistance^4^. It is used either in single or in a mixture of anti-diabetic medications. Adding PIO to MET and/or insulin secretagogues as part of triple oral therapy in patients with diabetes (type II) or case of binary drug failure is essential for reaching glycemic targets, improving β-cell function, and minimizing the risk factors involved in atherosclerosis^5^. PIO also improves glycated hemoglobin A1c (HbA1c) and fasting plasma glucose (FPG)^5^.

Glimepiride (GLM) (Fig. S1c) is a long-acting oral anti-diabetic used for decreasing the sugar level in blood^6^. GLM is used only for the treatment of diabetes type II. GLM may be used with Insulin or other drugs to obtain improved control over the blood sugar levels^6^.

Different analytical approaches were reported for the estimation of MET^7–11^, PIO^12–16^, or GLM^17–21^ alone and in combinations (MET and PIO)^22–27^, (MET and GLM)^28–30^ and (PIO and GLM)^31,32^.

Tribet-1 and Tribet-2 tablets are composed of (500 mg MET, 15 mg PIO, and 1 mg GLM) and (500 mg MET, 15 mg PIO, and 2 mg GLM), respectively^33^. These agents are effective for patients who require multiple agents to lower their blood glucose levels.

Chromatographic methods are well known for their superiority in separating and quantifying components in their complex mixtures^34^. Hyphenated chromatographic methods have an add-on advantage of enhancing method sensitivity and selectivity by using advanced spectroscopic techniques to detect and quantify components^35–37^. Several analytical methods have been reported for the simultaneous analysis of this triple antidiabetic mixture, including RP-HPLC methods^33,38–41^, LC–MS–MS^30^, and HPTLC^42^.

The reported RP-HPLC methods^33,38–41^ had some drawbacks, including; questionable methodologies and/or lack of satisfactory validation parameters. The reported method^33^ missed some validation parameters (LOD and LOQ) as well as system suitability parameters (resolution), in addition to very low MET (NTP)^33^. Reported methods^38,40^ had high-speed separation and short run-time incompatible with reported resolution values^38,40^. Also, the reported method^37^ did not identify the full details of the regression analysis^38^. Reported methods^33,38,39^ did not focus on either greenness assessment or the biological sample applicability. In the reported method^39^, the peak of MET appeared before the plasma peak, and the retention time of the plasma peak in spiked samples did not match that of the blank plasma chromatogram. None of the reported methods^33,38–41^ used IS in their calculations.

AQbD is a risk-assessment based and systematic method intended to find and reduce the variability sources that may lead to poor robustness of the analytical method, and confirm that the method meets its intended performance requirements^43^. In the Analytical quality by design models (AQbD), the “design space” is based on the intended purpose of the developed analytical method that allows its performance with allowable changes.

The current AQbD approach depends on the study of quality target product profile (QTPP), analytical target profile (ATP), and risk assessment tool for factors or critical method parameters (CMPs) that affect the method performance^43^. The (ATP) was to establish and validate robust, sensitive, and green RP-HPLC technique. Determination of the (ATP), critical quality attributes (CQAs), and critical method parameters (CMPs) is one of the essential steps in developing methods. Ishikawa diagrams as risk assessment tools can help identify the impact of different CMPs on the CQAs.

Design of Experiments (DOE) uses multivariate statistical techniques with advantages, such as the decrease in the total number of experimental runs needed DOE permits the establishment of mathematical models used to assess the statistical significance of different effects among many trivial parameters to determine the vital few ones^44^.

This paper represents the first HPLC method for the simultaneous determination of cited drugs based on the merits of the AQbD technique during development and optimization. Thus, the proposed method outperforms previously reported methods for determining the studied triple mixture, particularly for GLM concentration in a difficult dosage form ratio. (1:15:500) (GLM: PIO: MET). The paper focused on specifying the domain of the experimental space, where tolerance interval criteria for the studied chromatographic parameters intersect to obtain the method operable design region (MODR). The technique can be accounted for extending the method applications in biological samples by adding the internal standard to the analyzed compounds.

## Materials and method

### Materials and reagents

MET (99.00%), LIN (99.7%), PIO (99.5%), and GLM (99.7%). Excipients included microcrystalline hypromellose, cellulose, magnesium stearate, hydroxypropyl methylcellulose, pregelatinized starch, lactose monohydrate croscarmellose sodium, pregelatinized starch, and colloidal silicon dioxide. All the materials used in the experiment were gifts from Sigma for pharmaceutical industries (Moubarak Industrial Zone, Quesna-Menoufia-Egypt). Human plasma samples were kindly provided from the blood bank center of Tanta University Hospital after the required processes were done. All methods were carried out under relevant guidelines and regulations.

Egyptian markets dosage forms are Amaryl M 2/500 (2 mg Glimepiride and 500 mg Metformin) with Batch Number: 2/2024 from SANOFI AVENTIS-HANDOK Pharmaceuticals, Bioglita Plus (15/500) with Batch Number: 200061 produced by Al Andalous for Pharmaceutical Ind. (15 mg Pioglitazone and 500 mg Metformin) and Piompride 30/4, Batch Number: 191294, AVERROES PHARMA-Egypt (30 mg Pioglitazone and 4 mg Glimepiride).

Methanol of HPLC grade was purchased from (Fisher, UK). Potassium dihydrogen phosphate obtained from (Inter. Trade Co., Japan). Orthophosphoric acid of analytical grade was purchased from (Sigma-Aldrich, Germany). TEA of HPLC grade was purchased from (Oxford Laboratory, UK).

### Apparatus and HPLC software

Dionex UltiMate 3000 RS system (Thermo Scientific™, Dionex™, Sunnyvale, CA, USA) with RS auto-sampler injector, RS diode array detector, quaternary RS pump, and thermostated RS column compartment. ChromeleonR 7.1 software is used for data acquisition. Vortex (A & E, UK) and Hettich Centrifuge (Tuttlingen, Germany). A HANNA pH-meter (USA). Design-Expert version 11 software used for Design of Experiments (DOE).

### Chromatographic conditions

The CMPs qualified from the screening design were tested at different levels using the Box–Behnken optimization design. The values of the CAA were used to assess optimum chromatographic conditions by a mathematical technique using Derringer’s desirability algorithm within the predetermined MODR using the levels that best achieve the tolerance interval criteria for the studied chromatographic parameters. Separation was carried out by using methanol:0.05 M potassium dihydrogen phosphate buffer containing 0.05% triethylamine (78:22, v/v) as the mobile phase. The buffer pH was adjusted to pH 3.79 utilizing ortho-phosphoric acid. Detection at 227 nm using DAD. A 1.2 mL/min flow rate was used.

### Preparation of stock and working standard solutions

Stock solutions (1000 µg/mL) were prepared for the three drugs (MET, PIO, GLM) and for the internal standard (LIN) by weighing 100 mg of each, then transferred into four separate 100 mL volumetric flasks and diluted using methanol then stored at 4 °C in the refrigerator. Subsequently, suitable dilutions of each stock solution were made to prepare working standard solutions to obtain 50 µg/mL of MET, LIN, and PIO and 40 µg/mL of GLM using the mobile phase.

### Construction of calibration curves

#### Calibration in pure form

Different volumes of the previously prepared working standard solutions were transferred into separate 10 mL volumetric flasks with a constant volume of LIN (IS) (20 µL), and volumes were diluted using the mobile phase. Dilutions were made to attain solutions covering the dynamic working range 0.05–30.00 µg/mL PIO, 0.05–500.00 µg/mL MET, and 0.04–20.00 µg/mL GLM. 10 µL was injected from each solution, and the separation was made by using the previously mentioned separation conditions. The calibration curves were constructed by plotting the average peak area ratio to (0.1 µg/mL LIN) versus concentration, and the regression equations were computed.

#### Calibration in spiked human plasma

Different concentrations were prepared in spiked human plasma by using (50 µg/mL) working standard solutions of the internal standard (LIN) and the considered anti-diabetic drugs. Construction of calibration curves was made by plotting the average peak area ratio to (0.10 µg/mL LIN) against the corresponding concentrations of drug in spiked human plasma samples covering the dynamic working range of 0.04–2.00 µg/mL GLM and 0.05–2.00 µg/mL MET and PIO.

### Preparation of human plasma

Before the analysis, the frozen human plasma sample was permitted to be thawed and equilibrated to room temperature for about 1 h. Using multipulse vortex at 2000 rpm, the thawed plasma was vortexed for 30 s to confirm the well and homogenous mixing of the sample’s contents. In a centrifuge tube, an aliquot of 100 µL of blank plasma, a different aliquot from 50 µg/mL working standard solution of each drug. Obtained solutions were completed by using methanol up to 5 mL and vortexed at 2000 rpm twice to mix for 30 s to ensure the protein precipitation. Obtained plasma samples solutions were centrifuged for 30 min at 4000 rpm. From each supernatant, 1 mL was taken into a 5 mL volumetric flask, and the solutions were diluted using mobile phase to 5 mL. A cellulose acetate syringe filter (0.45 μm) was then used to filter all prepared solutions. An aliquot of 10 µL was injected from each solution at the before-stated separation conditions.

### Preparation of laboratory-prepared tablet

Tribet 2 XR tablets contain (2 mg GLM, 15 mg PIO and 500 mg MET) per tablet^33^ are not available in the Egyptian markets. Simulated synthetic tablets were prepared and used for analysis, regarding to preparation of laboratory prepared tablet^45^. The formula per five tablets was designed by weighing 2.5 g MET, 75 mg PIO and 10 mg GLM with the following excipients: 614.4 mg microcrystalline cellulose, 35 mg magnesium stearate, 75 mg hypromellose, 75 mg hydroxypropyl methylcellulose, 568 mg pregelatinized starch, 740 mg lactose monohydrate, 45 mg croscarmellose sodium, and 10 mg colloidal silicon dioxide. In a 100 mL volumetric flask, a weight equivalent to one tablet was transferred and dissolved with 70 mL methanol. The obtained solution was sonicated for 20 min, cooled, and completed to the mark by using the same solvent. The obtained solution was filtered, and the residues were washed. Serial dilutions were made to prepare different concentrations of the three drugs.

### Preparation of Egyptian-marketed dosage forms

Ten tablets of Amaryl M 2/500, Bioglita Plus, or Piompride Tablets were weighed, ground, and powdered in three separate mortars. Into separate 100 mL, volumetric flasks weight of powder equivalent to (500 mg MET and 2 mg GLM), (15 mg PIO and 500 mg MET) and (15 mg PIO and 2 mg GLM) were transferred and dissolved by using 75 mL methanol, respectively. The solutions were sonicated for 15 min, cooled, and completed up to the volume by using the same solvent. The solutions were filtered, then the residues were washed. Dilutions were made to achieve different concentrations of the two drugs through the three dosage forms.

### Analytical quality-by-design

The first step in the AQbD method was to determine the (QTPP) of the final pharmaceutical product, and then the (ATP) was identified based on the before-determined (QTPP). Subsequently, the determination of (CQAs) depends on initial trials and literature review.

### AQbD-based risk assessment using screening design

Risk analysis was performed to outline and determine the CQAs that might affect the method's efficiency and performance. Ishikawa diagram as a risk assessment tool can help define the impact of different critical method parameters CMPs on the CQAs^46^.

This paper aims to separate and analyze the three anti-diabetic drugs with optimum resolution and selectivity and minimum run time without interference from endogenous matrix compounds.

Fishbone or Ishikawa diagram was drawn to determine the significant parameters (CMPs) that affect the RP-HPLC method performance. Ishikawa diagram shows different factors that could be considered (column temperature, column length, flow rate, type of organic solvent, percentage of organic solvent, injection volume, detector, buffer type, buffer concentration, and buffer pH). Subsequently, preliminary trials were conducted to select the highly critical factors that would be included in the screening design (next step).

Five factors were qualified most prominently affecting the method performance (flow rate, percentage of methanol (%MeOH), column temperature, buffer pH, and buffer conc.).

Screening is a critical stage in AQbD to characterize the critical or significant factors before moving towards optimization design. Full factorial design for five factors of two levels for the screening phase will result in 2^5^ = 32 experiments (huge number), so; fractional factorial design (FFD) with resolution V (2^5–1^ = 16 experiments) was carried out to decrease the number of trials during the optimization and the development of an analytical method to characterize the influence of different CMP on the selected CQAs. The regression coefficients of the studied CMPs were determined by using a mathematical model obtained from the design consisting of main and possible interaction effects (Eq. 1) for each of the following:

Five responses or (CQA): Resolution-1 between MET and LIN (Rs-1), capacity factor-1 of MET (K′1), Resolution-3 between PIO and GLM (Rs-3), capacity factor-4 of GLM (K′4) and MET asymmetry (assym-MET).

1 $$\mathrm{Y}= {\upbeta }_{0}+ \sum_{\mathrm{i}=1}^{\mathrm{n}}{\upbeta }_{1}{\mathrm{X}}_{\mathrm{i}}+ \sum_{\mathrm{i}=1}^{\mathrm{n}}{\sum_{\mathrm{j}=\mathrm{i}+1}^{\mathrm{n}}\upbeta }{_\mathrm{ij}}{\mathrm{X}}_{\mathrm{i}}{\mathrm{X}}_{\mathrm{j}}+\upvarepsilon$$

where β_0_, βi, and βij represent the coefficients for each main and interaction effect, n is the number of CMPs, X is the examined factor, Y is the response measured, and ε represents the model residuals.

### AQbD method optimization with Box–Behnken design

The insignificant factors would be overlooked and kept constant during the optimization. The most favorable levels of CMPs obtained from the screening design were determined by further optimization utilizing response surface methodology. The three significant CMPs (buffer pH, flow rate, and % MeOH) were optimized by Box–Behnken Design with three levels to detect the most favorable levels of each parameter. The design was composed of a total of 17 experiments (5 centers + 12 non-center) to consider the experimental errors. The optimization procedure relied on Six CQAs named: resolution-1 (Rs-1: MET and LIN), capacity factor-1 (K′1), resolution-3 (Rs-3: PIO and GLM) & capacity factor-4 (K′4), number of theoretical plates of MET (NTP-MET) and GLM (NTP-GLM).

### Establishment of the method operable design region (MODR)

The MODR was determined based on the regression models and using the same software with an estimate of the probability of failure. All the criteria stated in the ATP within the design region are fulfilled.

Based on the CQAs tolerance interval (TI) with the suitable Sigma (S) and acceptable delta (d), the designated CQAs were predicted and plotted with the proportion of 0.90 (one-sided) and probability (α) = 0.05. The domain of the experimental space that intersects tolerance interval criteria (TI) was defined as the MODR of the established HPLC approach. Derringer’s desirability algorithm models applied to suggest the most optimum levels of each CMP depend on the definite optimization criteria.

## Results and discussion

### Analytical quality-by-design paradigm

QTPP determination was based on the delivery system, route of administration, dosage form type, and stability of studied drugs should be taken into consideration^47,48^. ATP was identified depending on the determined QTTP to obtain a more efficient RP-HPLC analytical technique able to identify and determine all APIs within an acceptable range (98–102)%, suitable retention times, symmetrical and sharp peaks, and reasonable specificity. The selection of (CQAs) was made depending on preliminary trials and a review of the literature.

### AQbD-based risk assessment using screening design

Risk assessment relied on the fishbone or Ishikawa diagram Fig. S2 that was constructed considering earlier scientific knowledge and preliminary trials. Preliminary studies were performed by trying different flow rates, columns, aqueous phase, proportions of the mobile phase, and organic modifiers. Based on the results of preliminary trials, there were some problems regarding peak asymmetry of (MET) and the resolution between (MET & LIN) and (PIO & GLM). The method's sensitivity to GLM of the lowest concentration in the analyzed dosage form was required to be considered. Peak asymmetry is strongly influenced by the pH of the buffer, column temperature, type, and organic modifier percent, while the flow rate could affect peaks resolution, shape, and area.

A list of the most critical parameters noted through the preliminary trials was used as factors or CMPs for the screening design (A: % MeOH, B: Flow rate, C: column temperature, D: buffer pH, and E: buffer concentration). The screening phase was based on five CQAs: Rs-1, Rs-3, K′-1, K′-4, and Asym-MET.

Due to the large variability of the analyzed triplet dosage form components and the binary ones (500 mg MET, 15 mg PIO, 2 mg GLM), a compromise was needed when selecting the detection wavelength using the DAD detector. The wavelength 227 nm was selected where GLM signal intensity was at the maximum. In contrast, MET signal intensity was low to allow the simultaneous detection of both drugs (MET & GLM) at this ratio. LIN was selected as IS of choice; the concentration 0.1 µg/mL was used for the plasma and 5 µg/mL for the separation and analysis in the pure form.

### Analysis of the experimental screening results

The procedures that have been followed to determine the CMPs that significantly affected each response (CQA):

1. Inspection of Pareto chart, half normal probability plots, then selection of the significant model terms.
2. Inspection of fitting statistics (R^2^ and adjusted R^2^).
3. ANOVA interpretation with Inspection of model residuals and factor significance.
4. Prediction equation coefficients interpretation based on sign and magnitude.
5. Inspection of the integrity of ANOVA diagnostics plots.
6. Characterization of significant factor performance based on the developed model graphs.

A half-normal probability plot is a graphical tool that uses these ordered estimated effects to help assess which factors are essential and which are unimportant. It displays the absolute values of the standardized |effect| from largest to smallest. The estimated |effect| of an insignificant factor was assigned to those on or close to the zero line, while the estimated |effect| of an essential factor was assigned to the ones off the line. Subsequently, the confirmation by the magnitude of F-value and corresponding p-value from ANOVA results and prediction equation coefficients' magnitude and sign. The positive sign of each parameter coefficient indicated that an effect of the parameter favors the response, while a negative sign suggested an inverse relationship between the parameter and the response. Table S1 represents the experimental results of the 16 fractional factorial design screening experiments. ANOVA results calculated for each response, such as p-value along with estimated responses’ coefficients greater than 0.9, were presented in Table 1, and the following was concluded:

**Table 1:** Coefficients and ANOVA statistical analysis for the five studied factors of the screening design.

	Intercept	A	B	C	D	E	AD
Rs-1	2.828	− 0.540		− 0.091	0.135		
p-values		< 1.00E−04		2.00E−04	< 1.00E−04		
Rs-3	4.028	− 0.593		− 0.253	− 1.569		1.227
p-values		< 1.00E−04		0.002	< 1.00E−04		< 1.00E−04
Assym-MET	1.419	0.114			0.108	− 0.054	
p-values		4.00E−04			6.00E−04	3.78E−02	
K1	1.642	0.021	− 0.238				
p-values		0.004	< 1.00E−04				
K-4	4.270	− 1.368	− 0.539	− 0.438	− 1.020		
p-values		< 1.00E−04	4.00E−04	1.40E−03	< 1.00E−04		

Rs: resolution, Assym: asymmetry; K′: capacity factor; A: %MeOH; B: flow rate; c: buffer pH; buffer pH and E: buffer concentration.

**Figure 1:**
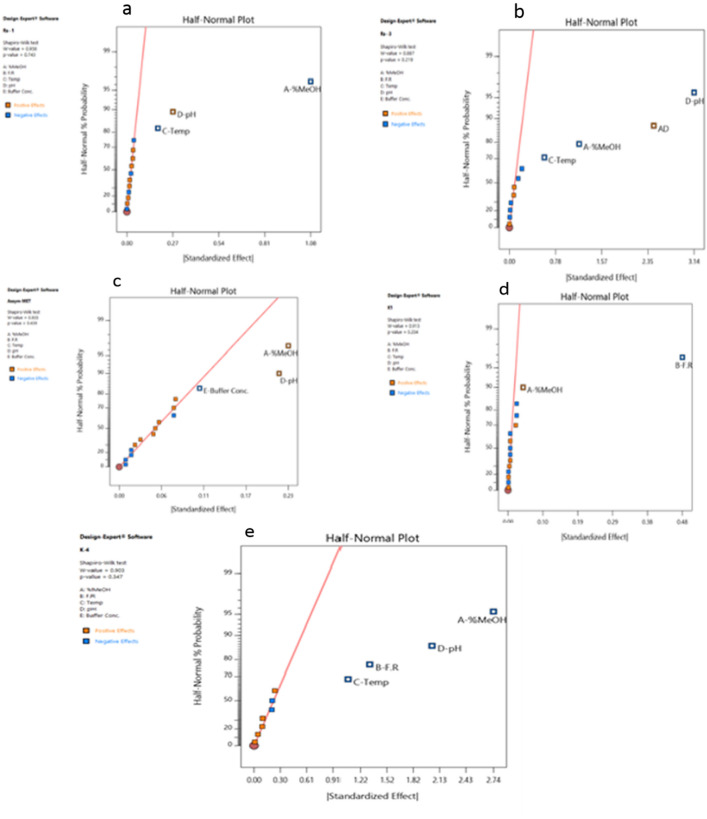
Half Normal probability plots fractional factorial design (FFD).
- Resolution-3 between (PIO and GLM) peaks (Rs-3):
  (Rs-3) was strongly affected by the buffer pH (D), %MeOH (A), and column temperature (C) with the negative effect of all. So, increasing the buffer pH up to pH 5 led to a sharp decrease in the Rs-3 value. There was a factors interaction between factors (A&D) (Fig. 1b).
  Further characterization of CMPs impacted on each CQA was done by the inspection of interaction plots that are very helpful tool to qualify the important parameters and selecting the suitable constant levels for the excluded ones. By the inspection of interaction plot (A&D) (Fig. S3), the Rs-3 value higher than 2 could be achieved by buffer pH (3) with MeOH% of 78%.
- MET asymmetry (Assym-MET):
  MET asymmetry was positively affected by both %MeOH (A) and buffer pH (D). So, decreasing MeOH% (A) or buffer pH (D) led to a decrease in MET asymmetry.
  MET asymmetry was the only response that was affected by the buffer concentration (E) with a negative effect. So, to decrease the MET asymmetry value, the buffer concentration should be used at the high level of 0.05 M. Thus, a decision was made to keep the buffer concentration (E) constant at (0.05 M) during the method optimization step (Fig. 1c).
- Capacity Factor-1 (K′-1):
  (K′-1) was strongly and negatively affected by the flow rate (B). (K′-1) also was positively affected by MeOH% (A) but to a very small extent compared to the effect of the flow rate (B) (Fig. 1d).
- Capacity Factor-4 (K′-4):
  K′-4 was negatively affected by %MeOH (A), buffer pH (D), flow rate (B), and temperature (C), with different magnitudes (Fig. 1e).
  After the Inspection of the screening design outcomes, the vital few factors to be optimized (A: MeOH %), (B: flow rate), and (D: buffer pH), were qualified for the optimization step due to their stronger effects. On the other hand, factors (E: buffer concentration) and (C: column temperature) were held constant at 0.05 M and 25 °C, respectively. A low-temperature setting will allow a greener separation procedure, and a high buffer concentration is essential to control MET peak asymmetry. The need for an optimization strategy arose as a result of the variable factor setting's requirement to improve each measured response individually, implying non-linearity, which is better described by using three-level response surface optimization designs.

- Resolution-1 between (MET & LIN) peaks (Rs-1):
  (Rs-1) was affected by three factors (A, C & D); MeOH% (A) had the most important and significant effect, while temperature (C) had the least effect. Increasing A and C decreased (Rs-1). (Fig. 1a). On the contrary, decreasing (D) decreased (Rs-1).

### (MODR) and optimization via Box–Behnken design

The AQbD approach's purpose is to define and outline the (MODR) which is a multidimensional combination and interaction of input variables and process factors that have been established to ensure the method quality^47^. In other words, it’s the region of (CMPs) that meet the (CQAs). Using the DoE strategy, the initial knowledge space was explored, and MODR was determined where the criteria stated in the ATP are met at a definite risk level^47^.

A Box–Behnken design was chosen to assess the influences of the three qualified CMPs (%MeOH, flow rate, and buffer pH) on the selected CQAs (Table S2). By the screening phase, we noticed in run No. 9 (Table S1 and Fig. S4) that severe overlap of the last two peaks took place when using the upper levels of the five factors. Box–Behnken design with 17 runs (Table S2) was more suitable, because it avoids the combination of the upper levels of all factors simultaneously and that fitted our optimization purpose.

**Figure 2:**
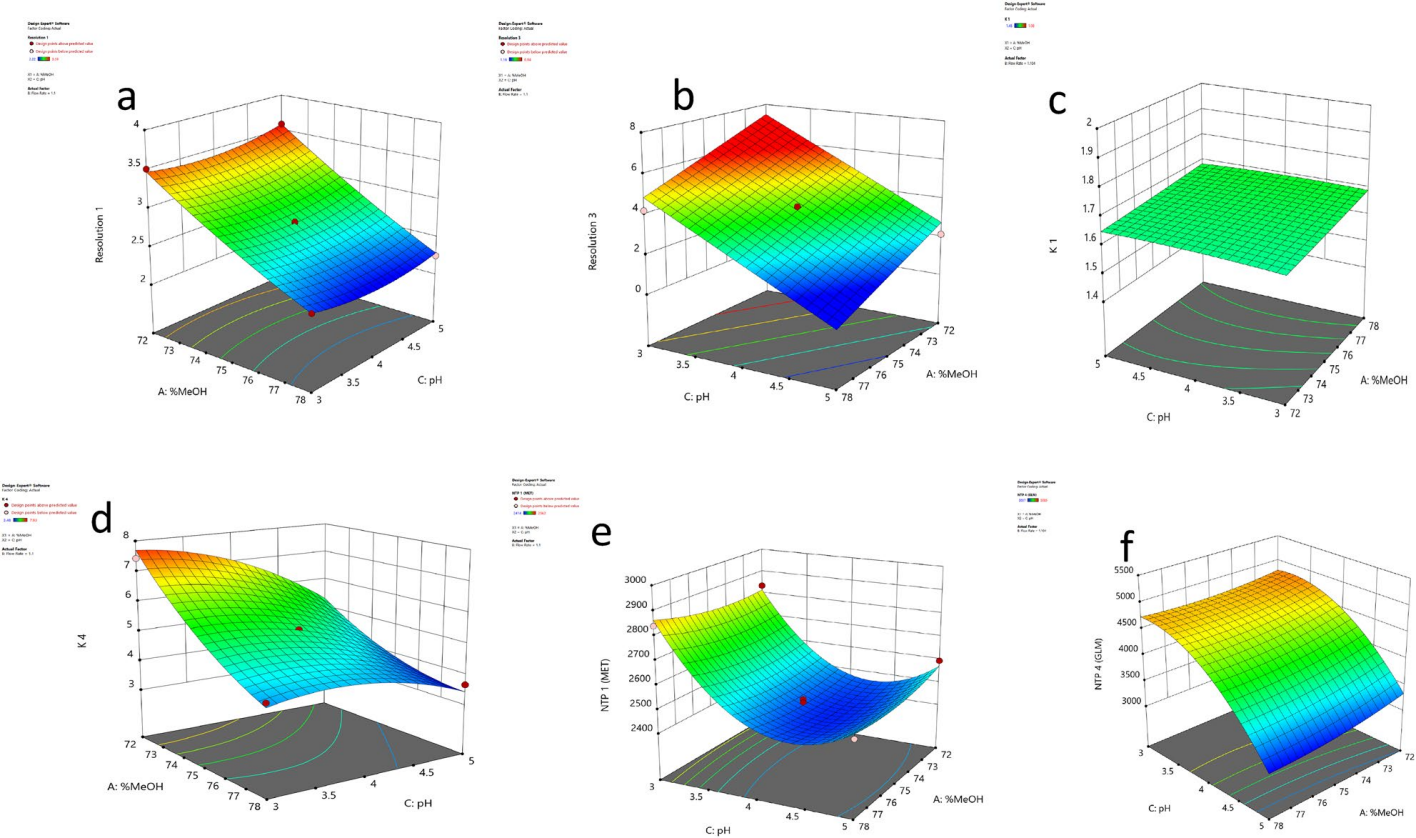
Response surfaces Box–Behnken design (BBD) for factor interaction.

All the developed models were quadratic, and variables behaved nonlinearly; this can be indicated by higher-order terms (x^2^). Also, models displayed high adjusted R^2^ and R^2^ values of more than 0.9, as shown in (Table S2) and insignificant Lack-of-fit relative to pure error values, where all indicated good model fitting. By the Inspection of the obtained model coefficients (Table 2) and 3D response surfaces (Fig. 2a–f).

- *(Rs-1) between (MET & LIN) peaks:* Fig. 2a shows a decrease in (Rs-1) value observed upon decreasing % MeO. (Rs-1) values between (2.2–3) were achieved using % MeOH not less than 74% with minimal effect of pH.
- *(Rs-3) between (PIO & GLM) peaks:* Fig. 2b shows a decrease in (Rs-3) values upon increasing pH and % MeOH. (Rs-3) values between (2.2–3.5) were achieved using %MeOH between (74–77) % and pH between (3–4).
- *Capacity Factor-1 (K′-1):* Fig. 2c shows that lower (K′-1) values were not obtained by variations in % MeOH and pH. However, (K′-1) value was significantly affected by the flow rate adopted in the analysis.
- *Capacity Factor-4 (K′-4):* Fig. 2d shows a decrease in (K′-4) values upon increasing %MeOH and to slight extent at higher pH values. Minimal (K′-4) values were obtained using % MeOH closer to 78% with slight effect when using pH between 3–5.
- *NTPs (MET) and (GLM):* Fig. 2e,f shows that higher NTPs of MET and GLM were achieved upon using pH values closer to 3 with minimal effect of % MeOH.

**Table 2:** Coefficients and ANOVA Statistical analysis for the three studied factors of the optimization design.

	Intercept	A	B	C	AB	AC	BC	A^2^	B^2^	C^2^
Resolution 1	2.750	− 0.616	− 0.058	0.024	0.018	− 0.035	− 0.018	0.053	0.056	0.108
p-values		< 1.00E−04	0.001	0.073	0.309	0.065	0.310	0.011	0.009	2.00E−04
Resolution 3	3.810	− 1.303		− 2.344						
p-values		< 1.00E−04		< 1.00E−04						
K′ 1	1.680	0.024	− 0.241	0.003	− 0.008	− 0.005	4.91E−19	0.001	0.026	− 0.006
p-values		< 1.00E−04	< 1.00E−04	0.296	0.048	0.155	1.000	0.695	< 1.00E−04	0.080
K′ 4	5.250	− 1.364	− 0.551	− 0.773		0.425		0.392		− 0.505
p-values		< 1.00E−04	< 1.00E−04	< 1.00E−04		0.002		0.003		6.00E−04
NTP 1 (MET)	2456.400	4.250	− 102.750	− 142.750	9.750	− 20.250	− 22.750	28.425	56.925	212.425
p-values		0.719	< 1.00E−04	< 1.00E−04	0.562	0.247	0.199	0.112	0.008	< 1.00E−04
NTP 4 (GLM)	4370.200	− 94.187	− 221.125	− 799.938	− 22.250	− 58.625	77.500	78.838	110.212	− 467.413
p-values		0.021	1.00E−04	< 1.00E−04	0.551	0.284	0.070	0.087	0.029	< 1.00E−04

K′: capacity factor; NTP: number of theoretical plates.

Increasing the GLM (NTP) and selecting of the suitable detection wavelength were successful measures that led to increasing method sensitivity to GLM with a more symmetrical and sharper peak of GLM. The buffer pH has a quadratic effect on the NTPs of MET and GLM, and this effect can’t be determined with other one factor at the time (OFAT) methods. From the design results, the best buffer pH that maximizes the NTPs and gives reasonable Rs values and capacity factor were selected at (3.7). This pH value led to a change in the ionization of both drugs and maximized the NTPs based on the pKa values of MET and GLM, which are 11.5 and 6.2, respectively.

To summarize the results of the optimization, process, the most significant factor was the % MeOH (A), as it was affecting nearly all responses; in most cases, the MeOH% needed to be increased. Flow rate (B) strongly affects the capacity factors (MET-K′-1) and (GLM-K′-4). Using (1.2 mL/min) flow rate led to the minimum K′ for MET & GLM. Buffer pH (C) strongly affected the NTPs (MET & GLM) and the Rs-3 between PIO and GLM.

Optimization criteria would help select the optimum levels of different CMPs. To optimize the different CQAs for optimum method efficiency and performance for the analysis of the three drugs, the following criteria were depicted:

1. To minimize Rs-1 and R-3 in range (2.2–3) and (2.2–3.5), respectively.
2. To minimize K′-1 and K′-4 for fast elution and minimum run time.
3. To maximize the NTPs for both MET and GLM.

Desirability plots Fig. S5a–c shows that to achieve the maximum desirability, the MeOH % should be as high as (76–78) % and pH should be between (3.5–4) as well as the flow rate should be at the maximum (1.2 mL/min).

The design region was generated by applying limitations (max & min) which were achieved (Rs-1) below 3.6, (Rs-3) below 4.5, (K′-1) below 1.98, (K′-4) below 5.5, NTP MET below 3000 and NTP GLM below 5500 with outcome proportion that achieves the (TI) of 0.9 (one-sided) as shown in Table S2.

The method operable design regions (overlay plots) illustrated in Fig. S5d–f showed that the optimum and best conditions could be obtained using higher MeOH ratio (76–78) %, pH should be between (3.6–5) and flow rate should be between (1.05–1.2) mL/min.

**Figure 3:**
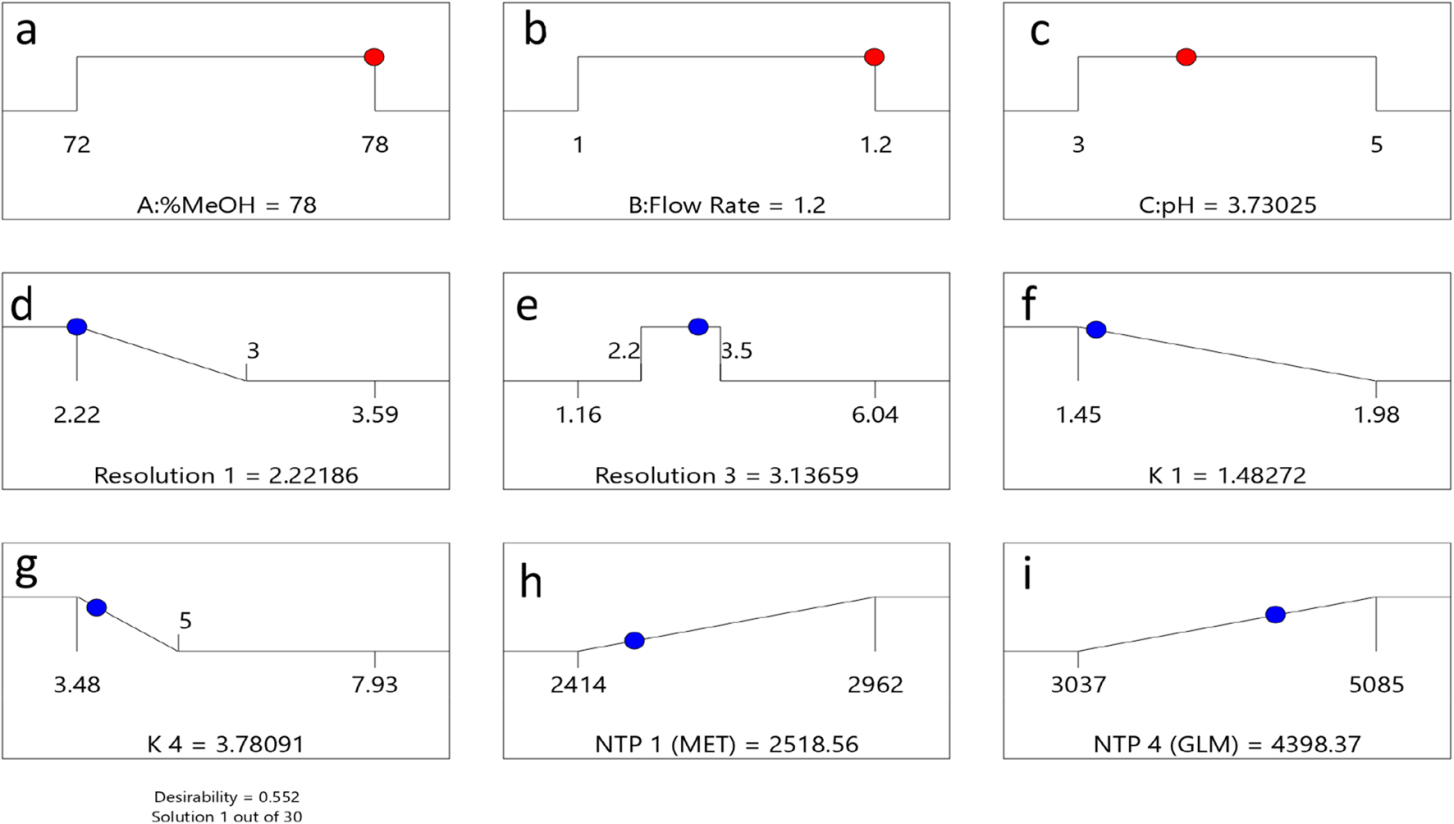
Solution ramps for optimum conditions (**a**–**i**).

**Figure 4:**
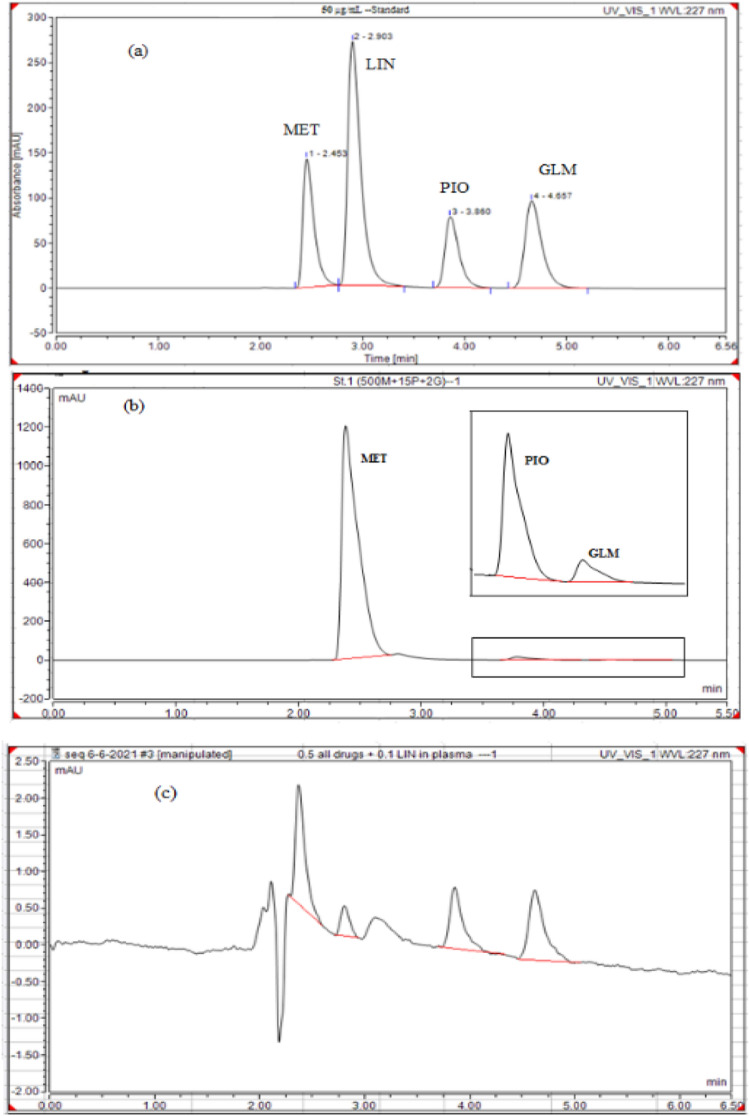
Chromatograms for standard pure drugs (**a**), lab-prepared mixture (**b**), and spiked plasma (**c**).

Using derringer’s desirability algorithm, 30 solutions resulted for the selected criteria; the optimum chromatographic parameters were proposed to be %MeOH(A) (78%) as shown in Fig. 3a, flow rate(B) (1.2 mL/min) as shown in Fig. 3b and buffer pH (3.73) as shown in Fig. 3c with expected attribute values of Rs-1 (2.22) as shown in Fig. 3d, Rs-3 (3.14) as shown in Fig. 3e, K′-1 (1.48) as shown in Fig. 3f, K′-4 (3.78) as shown in Fig. 3g, NTP (MET) (2518) as shown in Fig. 3h and NTP (GLM):(4389) as shown in Fig. 3i with a desirability value of (0.552). Desirability plots are shown in Fig. S5d–f.

These suggested optimum chromatographic conditions were verified and tested three times, and the mean of observed values were Rs-1 (2.28), Rs-3 (3.42), K′-1 (1.49), K′-4 (4.08), NTP (MET) (2568) and NTP (GLM): (4520). The predicted values were compared with those observed ones to demonstrate model predictability. All the results were satisfactory, with low prediction errors.

Finally, 78:22% MeOH: Phosphate buffer 0.05 M containing (0.05 v/v % triethylamine) pH (3.73) was the optimum mobile phase. 25 °C column temperature, 1.2 mL/min flow rate, and the PDA detector were set at 227 nm to allow detection of the three considered drugs.

### Method validation

The proposed AQbD technique was validated regarding ICH guidelines^49^. The results of system suitability parameter values at optimum separation conditions are presented in Table S3.

#### Linearity and range

The established RP-HPLC technique was used over the ranges of 0.05–30 µg/mL for PIO, 0.05–500 µg/mL for MET, and 0.04–20 µg/mL for GLM, as presented in Table 3.

**Table 3:** Regression parameters for estimation of MET, PIO and GLM in pure and tablet form using the developed method.

Drug	MET	PIO	GLM
Concentration range (µg/mL)	0.050–500.000	0.050–30.000	0.040–20.000
r	0.9999	0.9999	0.9999
a	− 0.057	0.115	0.198
b	2.990	1.677	2.292
Sa	0.041	0.014	0.010
Sb	0.000	0.018	0.001
S(y/x)	0.116	0.031	0.027
LOD	0.045	0.028	0.015
LOQ	0.136	0.085	0.044

r: correlation coefficient; b: slope; a: intercept; S_b_: standard deviation of slope; S_a_: standard deviation of intercept; S_y/x_: residual standard deviation; LOQ: limit of quantitation; LOD: limit of detection.

#### Limit of detection and quantitation (LOD and LOQ)

The detection limit and the quantitation limit (LOD and LOQ) were determined by referring to Eqs. (2) and (3); respectively, Table 3 shows LOD and LOQ.

2 $${\text{LOQ }} = { 1}0{\text{ S}}_{{\text{a}}} {\text{/b}}$$

3 $${\text{LOD }} = { 3}.{\text{3 S}}_{{\text{a}}} {\text{/b}}$$

where b is the slope of the calibration curve and S_a_ is the standard deviation of the y-intercept of regression lines.

#### Accuracy

The accuracy of the proposed approach was determined by calculating the mean % recoveries at three different concentration levels (triplicate determination) for MET (500, 400 and 250) µg/mL, PIO (15, 12, and 7.5) µg/mL, and GLM (2, 1.6 and 1) µg/mL (Table S4).

#### Precision

##### Intra-day precision

Intra-day precision was assessed by using three replicate analyses at three drug concentration levels on the same day. The (SD) and (% RSD) were calculated for the results of the analysis as presented in (Table S5).

##### Inter-day precision

The same three concentration levels of each drug were analyzed in three replicates at different three successive days. The (SD) and (% RSD) were calculated for the results of the analysis as presented in (Table S5).

All results were less than 2, as presented in (Table S5) demonstrating that the technique was precise.

#### Robustness

According to ICH guidelines^49^ the robustness of an analytical process is the ability of method performance to remain unaffected by small but deliberate changes. Defining MODR based on the AQbD approach aid in assessing the robustness and ruggedness of the analytical method before validation, as the MODR itself is the region in which the CMPs meet the CQAs.

Robustness studies of the established RP-HPLC using the AQbD technique were carried out by using the multivariate design-based approach to study the effect of simultaneous variation of the studied five factors (pH, methanol%, flow rate, temperature, and buffer concentration) on the selected responses. A regular two levels (− 1, + 1) factorial screening design of eight runs with five factors was used for robustness testing (Table S6) to study only the main effects of the proposed study parameters (where factor interactions are not common, and to reduce experimentation time) on HPLC method performance.

Small changes in studied factors were carried out. The inspection of pareto charts revealed that the effect of all the considered parameters (CMPs) were non-significant on the pre-selected responses (CQAs) this was confirmed by that all experimental t-values were lower than the critical t-values limit as shown in Fig. S6a–g. The results indicate good stability and chromatographic performance of the established approach to small deliberate changes in its (CMPs).

#### Specificity

The specificity of the established approach was demonstrated by comparing the test results and chromatograms of simulated tablet solution containing all excipients expected to be present in the dosage form and solution containing biological matrices of plasma with that of a standard solution of pure drugs of the same concentrations at the optimum separation conditions as presented in (Fig. 4a,b).

### Greenness evaluation method

For the estimation of the greenness of an analytical technique analytical Eco-Scale approach was applied^50^. The sum of the total penalty points was calculated for the whole procedure. According to the calculated results, the validated approach has acceptable greenness with an analytical eco-scale score 73 (Table S7).

### Application to simulated prepared tablets

The validated approach was applied to simultaneously determine three different concentration levels of the three antidiabetic drugs in its laboratory-prepared tablets in the ratio (500:15:2) (MET:LIN:EMP). The %recovery, SD, and %RSD were calculated, and acceptable results were obtained (Table 4). The results of the validated approach for the three concentrations of the simulated prepared tablets were compared to those found by applying the published RP-HPLC technique using a t-test and F-test at a 95% confidence level regarding accuracy and precision, respectively. The calculated values did not exceed the tabulated ones, demonstrating any significant difference between the reported and the proposed methods, as presented in (Table 4).

**Table 4:** Comparison between the assay of prepared tablets using the proposed HPLC method and reported method.

Proposed method				Reported method		
Drugs	MET	PIO	GLM	MET	PIO	GLM
Mean (Ẋ)	100.804	100.099	99.657	100.209	99.731	100.398
S	0.389	0.344	0.115	1.326	0.432	1.260
%RSD	0.386	0.344	0.115	1.324	0.433	1.255
t_cal_	0.746	1.153	1.014	t_tab_		2.770
F_cal_	11.628	1.578	0.008	F_tab_		19.000

X: Mean of % recoveries; S.D: standard deviation; R.S.D: relative standard deviation; t_cal_: calculated t-value; t_tab_: tabulated t-value; F_cal_: calculated F-value; F_tab_: tabulated F-value.

### Application to Egyptian market products

The validated method was applied for the simultaneous determination of (MET & GLM) in *Amaryl M 2/500* tablets (2 mg Glimepiride and 500 mg Metformin) with Batch Number: 2/2024, (PIO & MET) in *Bioglita Plus* Tablets (15/500) with Batch Number: 200061 and (PIO & GLM) in *Piompride 30/4* tablets, Batch Number: 191294, AVERROES PHARMA-Egypt (30 mg Pioglitazone and 4 mg Glimepiride). The %recovery, SD, and %RSD were calculated, and acceptable results were obtained (Table S8).

### Results of analysis in spiked plasma samples

The validated method was applied for the simultaneous quantitation of (MET, PIO & GLM) with 0.1 µg/mL LIN internal standard in spiked plasma samples, as presented in (Fig. 4c) Calibration curves were plotted covering the range of 0.05–2 µg/mL of MET & PIO and 0.04–2 µg/mL of GLM. Results are presented in Table 5.

**Table 5:** Regression parameters for estimation of MET, PIO and GLM in spiked human plasma using the developed method.

Drug	MET	PIO	GLM
Concentration range (µg/mL)	0.050–2.000	0.050–2.000	0.040–2.000
r	0.9999	0.9999	0.9999
a	0.075	0.150	0.184
b	2.665	1.614	2.312
Sa	0.014	0.009	0.012
Sb	0.014	0.010	0.014
S(y/x)	0.022	0.017	0.024
LOD	0.017	0.019	0.017
LOQ	0.052	0.058	0.051

r: correlation coefficient; b: slope; a: intercept; S_b_: standard deviation of slope; S_a_: standard deviation of intercept; S_y/x_: residual standard deviation; LOQ: limit of quantitation; LOD: limit of detection.

## Conclusion

This study describes a fast, sensitive, and green RP-HPLC method that was optimized and validated by using the AQbD paradigm for the identification and estimation of MET, PIO, and GLM simultaneously in their pure and laboratory-prepared tablet. The method was extended to determine the studied drugs in spiked plasma samples. A scientifically organized approach was followed in developing, optimizing, and even in validating the proposed method, where MODR was assessed to minimize the number of out-of-trend results by determination of the design region where all the stated chromatographic criteria were satisfied. Because of the high level of quality built into this RP-HPLC method, it is an excellent candidate for routine analysis in quality control labs and bioanalytical analyses. Moreover, the procedures and details mentioned in this paper can help peer experimenters build and interpret AQbD-built methods efficiently.

## Supplementary Information

Supplementary Information.

## Data Availability

All data generated or analyzed during this study are included in this article.
