# Supplementary material for: Deduction of the operable design space of RP-HPLC technique for the simultaneous estimation of metformin, pioglitazone, and glimepiride
Source: Sci Rep. 2023 Mar 16;13:4334. doi: 10.1038/s41598-023-30051-x (PMC10020468; doi:10.1038/s41598-023-30051-x)
Supplement: Supplementary file 1 — Supplementary Information. [file 41598_2023_30051_MOESM1_ESM.docx]

**Table S1**: Factors and levels selected for screening phase studies

|  | Factor 1 | Factor 2 | Factor 3 | Factor 4 | Factor 5 | Response 1 | Response 2 | Response 3 | Response 4 | Response 5 |
| --- | --- | --- | --- | --- | --- | --- | --- | --- | --- | --- |
| Run | A: %MeOH | B: F.R | C: Temp | D: pH | E: Buffer Conc. | Rs-1 | Rs-3 | Asym-MET | K1 | K-4 |
|  | % | mL/min | C |  | M |  |  |  |  |  |
| 1.000 | 72.000 | 1.000 | 40.000 | 3.000 | 0.040 | 3.200 | 6.950 | 1.340 | 1.850 | 6.640 |
| 2.000 | 78.000 | 1.000 | 40.000 | 3.000 | 0.050 | 2.040 | 3.510 | 1.410 | 1.870 | 4.100 |
| 3.000 | 72.000 | 1.200 | 25.000 | 3.000 | 0.040 | 3.330 | 7.750 | 1.140 | 1.380 | 6.540 |
| 4.000 | 72.000 | 1.200 | 40.000 | 3.000 | 0.050 | 3.070 | 7.030 | 1.270 | 1.360 | 5.360 |
| 5.000 | 78.000 | 1.200 | 25.000 | 5.000 | 0.040 | 2.470 | 0.000 | 1.690 | 1.470 | 0.000 |
| 6.000 | 72.000 | 1.200 | 40.000 | 5.000 | 0.040 | 3.470 | 1.490 | 1.580 | 1.400 | 3.700 |
| 7.000 | 78.000 | 1.200 | 40.000 | 3.000 | 0.040 | 2.000 | 3.790 | 1.410 | 1.410 | 3.330 |
| 8.000 | 78.000 | 1.000 | 25.000 | 3.000 | 0.040 | 2.310 | 3.930 | 1.540 | 1.920 | 4.560 |
| 9.000 | 78.000 | 1.200 | 40.000 | 5.000 | 0.050 | 2.380 | 0.000 | 1.700 | 1.410 | 0.000 |
| 10.000 | 72.000 | 1.200 | 25.000 | 5.000 | 0.050 | 3.470 | 2.050 | 1.230 | 1.390 | 4.530 |
| 11.000 | 72.000 | 1.000 | 40.000 | 5.000 | 0.050 | 3.400 | 1.750 | 1.330 | 1.840 | 4.640 |
| 12.000 | 78.000 | 1.200 | 25.000 | 3.000 | 0.050 | 2.220 | 3.880 | 1.300 | 1.410 | 3.700 |
| 13.000 | 78.000 | 1.000 | 40.000 | 5.000 | 0.040 | 2.070 | 2.840 | 1.620 | 1.900 | 3.930 |
| 14.000 | 78.000 | 1.000 | 25.000 | 5.000 | 0.050 | 2.550 | 0.000 | 1.600 | 1.910 | 0.000 |
| 15.000 | 72.000 | 1.000 | 25.000 | 3.000 | 0.050 | 3.370 | 7.940 | 1.080 | 1.850 | 8.090 |
| 16.000 | 72.000 | 1.000 | 25.000 | 5.000 | 0.040 | 3.630 | 2.010 | 1.470 | 1.900 | 5.600 |

^*^N.A. not applicable

Rs: resolution, Asym: asymmetry, K': capacity factor

**Table S2:** Experimental runs and the obtained responses of the central composite design for optimization

|  | Factor 1 | Factor 2 | Factor 3 | Response 1 | Response 2 | Response 3 | Response 4 | Response 5 | Response 6 |
| --- | --- | --- | --- | --- | --- | --- | --- | --- | --- |
| Run | A: %MeOH | B: Flow Rate | C: pH | Resolution 1 | Resolution 3 | K' 1 | K' 4 | NTP 1 (MET) | NTP 4 (GLM) |
| 1 | 72.000 | 1.200 | 4.000 | 3.380 | 5.370 | 1.450 | 6.510 | 2414.000 | 4428.000 |
| 2 | 75.000 | 1.100 | 4.000 | 2.770 | 4.000 | 1.680 | 5.260 | 2468.000 | 4385.000 |
| 3 | 72.000 | 1.100 | 3.000 | 3.510 | 7.910 | 1.650 | 7.430 | 2835.000 | 4809.000 |
| 4 | 75.000 | 1.200 | 3.000 | 2.840 | 5.740 | 1.450 | 4.990 | 2780.000 | 4549.000 |
| 5 | 75.000 | 1.100 | 4.000 | 2.730 | 3.980 | 1.680 | 5.240 | 2425.000 | 4403.000 |
| 6 | 78.000 | 1.100 | 5.000 | 2.250 | 1.650 | 1.690 | 3.660 | 2519.000 | 3037.000 |
| 7 | 75.000 | 1.200 | 5.000 | 2.890 | 1.160 | 1.460 | 3.480 | 2444.000 | 3096.000 |
| 8 | 78.000 | 1.100 | 3.000 | 2.310 | 4.200 | 1.700 | 4.260 | 2840.000 | 4746.000 |
| 9 | 72.000 | 1.100 | 5.000 | 3.590 | 2.130 | 1.660 | 5.130 | 2595.000 | 4886.000 |
| 10 | 78.000 | 1.200 | 4.000 | 2.220 | 2.810 | 1.490 | 3.630 | 2486.000 | 4187.000 |
| 11 | 75.000 | 1.000 | 3.000 | 2.910 | 6.040 | 1.940 | 6.160 | 2962.000 | 5085.000 |
| 12 | 75.000 | 1.100 | 4.000 | 2.740 | 3.900 | 1.680 | 5.230 | 2438.000 | 4239.000 |
| 13 | 72.000 | 1.000 | 4.000 | 3.540 | 5.640 | 1.910 | 7.930 | 2617.000 | 4887.000 |
| 14 | 78.000 | 1.000 | 4.000 | 2.310 | 3.000 | 1.980 | 4.540 | 2650.000 | 4735.000 |
| 15 | 75.000 | 1.100 | 4.000 | 2.760 | 3.960 | 1.680 | 5.220 | 2468.000 | 4418.000 |
| 16 | 75.000 | 1.000 | 5.000 | 3.030 | 1.230 | 1.950 | 4.390 | 2717.000 | 3322.000 |
| 17 | 75.000 | 1.100 | 4.000 | 2.770 | 3.950 | 1.680 | 5.230 | 2483.000 | 4406.000 |
|  |  |  | R2 | 0.998 | 0.940 | 0.999 | 0.982 | 0.985 | 0.995 |
|  |  |  | Adjusted R**^2^** | 0.995 | 0.931 | 0.999 | 0.972 | 0.966 | 0.987 |
|  |  |  | d | 2.850 | 3.850 | 1.700 | 5.200 | 2600.000 | 4300.000 |
|  |  |  | s | 1.500 | 1.000 | 0.950 | 2.500 | 1200.000 | 1500.000 |
|  |  |  | FDS | 0.950 | 0.990 | 1.000 | 0.980 | 0.990 | 0.980 |
|  |  |  | T.I | 2.360 | 2.450 | 2.360 | 2.360 | 2.360 | 2.450 |

α = 0.05 (Probability), proportion= 0.95, d: acceptable delta, S: sigma, FDS: Fraction design space, K': capacity factor, NTP: number of theoretical plats,

T.I: tolerance interval multiplier

**Table S3.** System suitability parameters at optimum chromatographic conditions.

| Parameter | drug | results | Reference value |
| --- | --- | --- | --- |
| Retention time | MET | 2.453 | ---- |
|  | LIN | 2.903 |  |
|  | PIO | 3.860 |  |
|  | GLM | 4.667 |  |
| Tailing (T_f_) | MET | 1.220 | 0.800 ≤ T_f_ ≤ 1.200 |
|  | PIO | 1.170 |  |
|  | GLM | 1.150 |  |
| Number of theoretical plates (NTP) | MET | 2568.170 | ˃ 2000.000 |
|  | PIO | 4536.000 |  |
|  | GLM | 4520.490 |  |
| Resolution (R_s_) | MET-LIN | 2.283 | ˃ 1.500 |
|  | LIN-PIO | 4.320 |  |
|  | PIO-GLM | 3.428 |  |

**Table S4**: Results of accuracy for the determination of MET, PIO and GLM by the proposed HPLC method.

| Claimed Conc. (µg/ mL) | | | Proposed RP-HPLC | | | | | | Reported HPLC | | | | | |
| --- | --- | --- | --- | --- | --- | --- | --- | --- | --- | --- | --- | --- | --- | --- |
|  |  |  | MET | | PIO | | GLM | | MET | | PIO | | GLM | |
| MET | PIO | GLM | Obtained Conc. (µg/ mL) | Recovery % | Obtained Conc. (µg/ mL) | Recovery % | Obtained Conc. (µg/ mL) | Recovery % | Obtained Conc.  (µg/ mL) | Recovery % | Obtained Conc. (µg/ mL) | Recovery % | Obtained Conc. (µg/ mL) | Recovery % |
| 500.000 | 15.000 | 2.000 | 503.769 | 100.754 | 15.067 | 100.445 | 1.992 | 99.605 | 495.592 | 99.118 | 15.032 | 100.214 | 2.029 | 101.444 |
| 400.000 | 12.000 | 1.600 | 401.772 | 100.443 | 11.971 | 99.757 | 1.593 | 99.577 | 299.467 | 99.822 | 8.964 | 99.597 | 1.188 | 98.999 |
| 250.000 | 7.500 | 1.000 | 253.040 | 101.216 | 7.507 | 100.094 | 0.998 | 99.788 | 203.371 | 101.686 | 5.963 | 99.382 | 0.806 | 100.750 |
| Mean % | | | 100.804 | | 100.099 | | 99.657 | | 100.209 | | 99.731 | | 100.398 | |
| S.D. | | | 0.389 | | 0.344 | | 0.115 | | 1.326 | | 0.432 | | 1.260 | |
| RSD % | | | 0.386 | | 0.344 | | 0.115 | | 1.324 | | 0.433 | | 1.255 | |
| F _cal_ | | | 11.628 | | 1.578 | | 0.008 | |  | | F _tab_ | | 19.000 | |
| t_cal_ | | | 0.746 | | 1.153 | | 1.014 | |  | | t _tab_ | | 2.770 | |

S.D.: Standard deviation, RSD %: Percent relative standard deviation

**Table S5**: Results of intra-day and inter-day precision for determination of MET, PIO and GLM

| Concentration taken (µg/mL) | | Intra-day precision | | | Inter-day precision | | |
| --- | --- | --- | --- | --- | --- | --- | --- |
|  |  | Mean conc. found | S.D. | %RSD | Mean conc. found | S.D. | %RSD |
| MET | 500.000 | 504.668 | 0.491 | 0.097 | 504.563 | 1.135 | 0.225 |
|  | 400.000 | 406.914 | 0.575 | 0.141 | 405.536 | 1.269 | 0.313 |
|  | 250.000 | 253.040 | 1.512 | 0.598 | 252.883 | 0.137 | 0.054 |
| PIO | 15.000 | 15.067 | 0.106 | 0.701 | 15.125 | 0.051 | 0.339 |
|  | 12.000 | 11.807 | 0.042 | 0.359 | 12.019 | 0.065 | 0.540 |
|  | 7.500 | 7.471 | 0.074 | 0.984 | 7.538 | 0.031 | 0.408 |
| GLM | 2.000 | 1.996 | 0.010 | 0.499 | 1.996 | 0.004 | 0.212 |
|  | 1.600 | 1.610 | 0.014 | 0.885 | 1.598 | 0.010 | 0.653 |
|  | 1.000 | 1.003 | 0.008 | 0.773 | 0.997 | 0.006 | 0.586 |

*Mean for three determinations, S.D. Standard deviation, R.S.D: relative standard deviation.

**Table S6**: Studied factor and responses for evaluation of robustness

|  | Factor 1 | Factor 2 | Factor 3 | Factor 4 | Factor 5 | Response 1 | Response 2 | Response 3 | Response 4 | Response 5 | Response 6 | Response 7 |
| --- | --- | --- | --- | --- | --- | --- | --- | --- | --- | --- | --- | --- |
| Run | A: pH | B: Temp | C: Flow rate | D: %MeOH | E: Buffer Conc. | Rs1 | Rs 3 | NTP GLM | AUC MET | AUC LIN | AUC PIO | AUC GLM |
|  |  | °C | mL/min | % | mM |  |  |  |  |  |  |  |
| 4 | 3.880 | 24.000 | 1.180 | 77.600 | 49.000 | 2.630 | 3.600 | 5488.000 | 8.270 | 7.660 | 5.296 | 6.490 |
| 6 | 3.680 | 26.000 | 1.220 | 77.600 | 49.000 | 2.630 | 3.830 | 5766.000 | 8.028 | 7.533 | 5.093 | 6.292 |
| 3 | 3.680 | 26.000 | 1.180 | 77.600 | 51.000 | 2.610 | 3.820 | 5531.000 | 8.150 | 7.650 | 5.199 | 6.420 |
| 1 | 3.880 | 24.000 | 1.220 | 77.600 | 51.000 | 2.510 | 3.490 | 5258.000 | 7.430 | 7.086 | 4.779 | 5.865 |
| 2 | 3.680 | 24.000 | 1.180 | 78.400 | 51.000 | 2.500 | 3.270 | 5434.000 | 7.888 | 7.281 | 5.173 | 6.268 |
| 5 | 3.680 | 24.000 | 1.220 | 78.400 | 49.000 | 2.480 | 3.560 | 5518.000 | 7.978 | 7.515 | 5.156 | 6.361 |
| 7 | 3.880 | 26.000 | 1.180 | 78.400 | 49.000 | 2.460 | 3.560 | 5859.000 | 8.197 | 7.542 | 5.297 | 6.435 |
| 8 | 3.880 | 26.000 | 1.220 | 78.400 | 51.000 | 2.390 | 3.190 | 5498.000 | 7.617 | 6.987 | 4.825 | 5.910 |

**Table S7**: Results for calculation of analytical eco-scale total score

| **Reagents** | amount | Signal word | No. of pictograms | Sub-total PP |
| --- | --- | --- | --- | --- |
| 1- MeOH | <10ml = 1 | Danger=2 | 3.000 | 6.000 |
| 2- Pot. Dihydrogen phosphate | <10ml = 1 | 0 | 0.000 | 0.000 |
| 3- Phosphoric acid | <10ml = 1 | Danger=2 | 1.000 | 2.000 |
| 4- TEA (Triethylamine) | <10ml = 1 | Danger=2 | 3.000 | 6.000 |
| **Total PP for reagents** | | | | 14.000 |
| **Instrument** | HPLC=2 | | | 2.000 |
| Occupational hazard | Not emit vapors and gases = 0 | | | 0.000 |
| Waste | 1-10ml = 2 | | | 2.000 |
| Treatment of waste | No treatment(waste)= 3 | | | 3.000 |
| **Total PP for instruments** | | | | 7.000 |
| **Freezer -20 =2** | | | | 2.000 |
| **Centrifugation =2** | | | | 2.000 |
| **Vortex =2** | | | | 2.000 |
| **Total PP for instruments** | | | | 6.000 |
| Total PP | | | | 27.000 |
| Analytical Eco-Scale total score: | | | | **73.000** (acceptable green) |

>75 represents excellent green analysis,

>50 represents acceptable green analysis,

<50 represents inadequate green analysis.

**Table S8**: Results of assay of Amaryl M 2/500^®^, Bioglita Plus^®^ and Piompride^®^ tablets using the proposed HPLC method

| Proposed RP-HPLC (Amaryl M 2/500®) | | | | | | | | | | Proposed RP-HPLC (Bioglita Plus®) | | | | | | | | Proposed RP-HPLC (Piompride®) | | | | | | | | | | |  |
| --- | --- | --- | --- | --- | --- | --- | --- | --- | --- | --- | --- | --- | --- | --- | --- | --- | --- | --- | --- | --- | --- | --- | --- | --- | --- | --- | --- | --- | --- |
| Claimed Conc. (µg/ mL) | | MET | | | | GLM | | | | Claimed Conc. (µg/ mL) | | MET | | | | PIO | | Claimed Conc.  (µg/ mL) | | PIO | | | | GLM | | | | |  |
| MET | GLM | Found Conc. (µg/ mL) | Rec.% | | | Found Conc. (µg/ mL) | | Rec. % | | MET | PIO | Found Conc. (µg/ mL) | | | Rec. % | Found Conc. (µg/ mL) | Rec.% | PIO | GLM | Found Conc. (µg/ mL) | | Rec.% | | Found Conc. (µg/ mL) | | | Rec. % | |  |
| 500 | 2.00 | 502.288 | 100.458 | | | 1.996 | | 99.804 | | 500 | 15.00 | 507.678 | | | 101.536 | 15.135 | 100.900 | 15 | 2.0 | 14.944 | | 99.627 | | 2.001 | | | 100.059 | |  |
| 350 | 1.40 | 347.237 | 99.210 | | | 1.398 | | 99.883 | | 400 | 12.00 | 402.199 | | | 100.550 | 11.916 | 99.299 | 9 | 1.2 | 8.918 | | 99.093 | | 1.186 | | | 98.819 | |  |
| 250 | 1.00 | 254.227 | 101.691 | | | 0.995 | | 99.503 | | 300 | 9.00 | 302.309 | | | 100.770 | 8.991 | 99.900 | 7.5 | 1.0 | 7.517 | | 100.229 | | 1.010 | | | 101.021 | |  |
| Mean % | |  | |  | 100.453 |  |  | | 99.318 | | Mean % | | |  | 100.952 |  | 99.318 |  | Mean % |  |  | | 99.650 | |  |  | | 99.318 | |
| S.D. | |  | |  | 1.240 |  |  | | 0.340 | | S.D. | |  | | 0.517 |  | 0.340 |  | S.D. |  |  | | 0.569 | |  |  | | 0.340 | |
| RSD % | |  | |  | 1.234 |  |  | | 0.342 | | RSD % | |  | | 0.513 |  | 0.342 |  | RSD % |  |  | | 0.571 | |  |  | | 0.342 | |

S.D. Standard deviation, R.S.D: relative standard deviation

**Fig. S1:** Chemical structures of (a): Metformin (MET), (b): Pioglitazone (PIO), (c): Glimepride (GLM)

**Fig. S2**: Ishikawa diagram indicates the critical parameters that will be considered in method development and optimization.

**Fig. S3:** Interaction plot fractional factorial design (FFD).

**Fig. S4:** Chromatogram (run No. 9) showing sever overlap of the last two peaks (PIO & GLM) when using the upper levels of all factors.

**Fig. S5:** Desirability and overlay plots

**Fig. S6:** Pareto charts of robustness studies


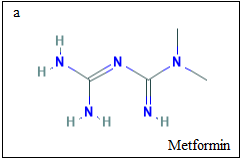

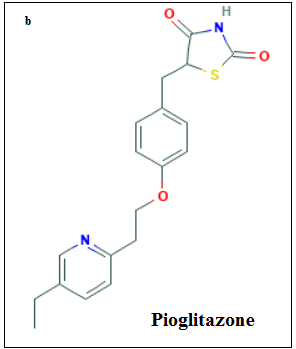

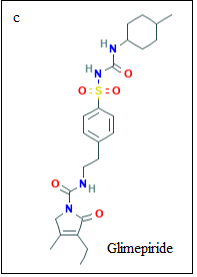


**Fig. S1:** Chemical structures of (a): Metformin (MET), (b): Pioglitazone (PIO), (c): Glimepride (GLM)


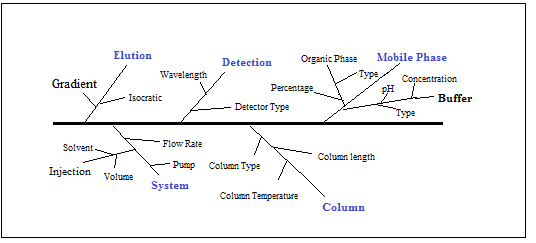


**Fig. S2**: Ishikawa diagram indicates the critical parameters that will be considered in method development and optimization.


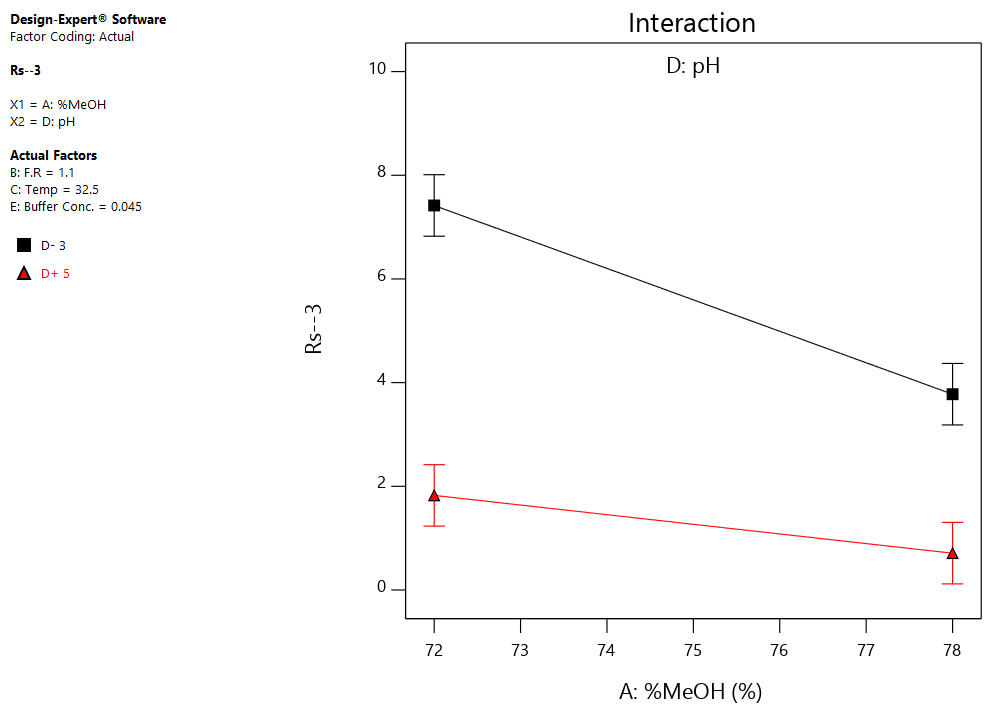


**Fig. S3:** Interaction plot fractional factorial design (FFD)


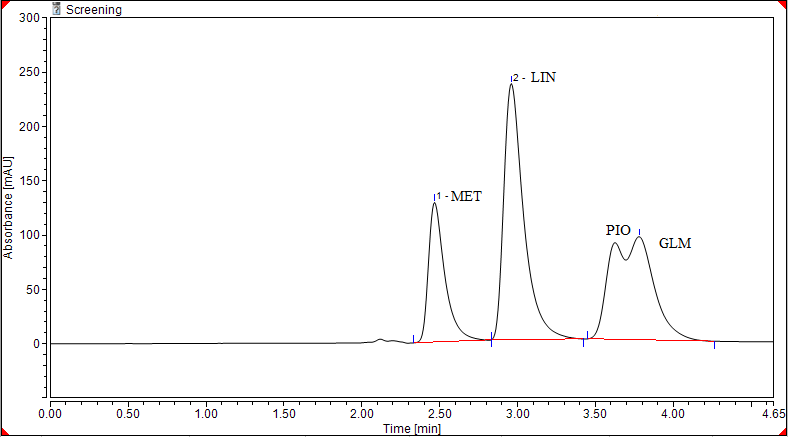


**Fig. S4:** Chromatogram (run No. 9) showing sever overlap of the last two peaks (PIO & GLM) when using the upper levels of all factors


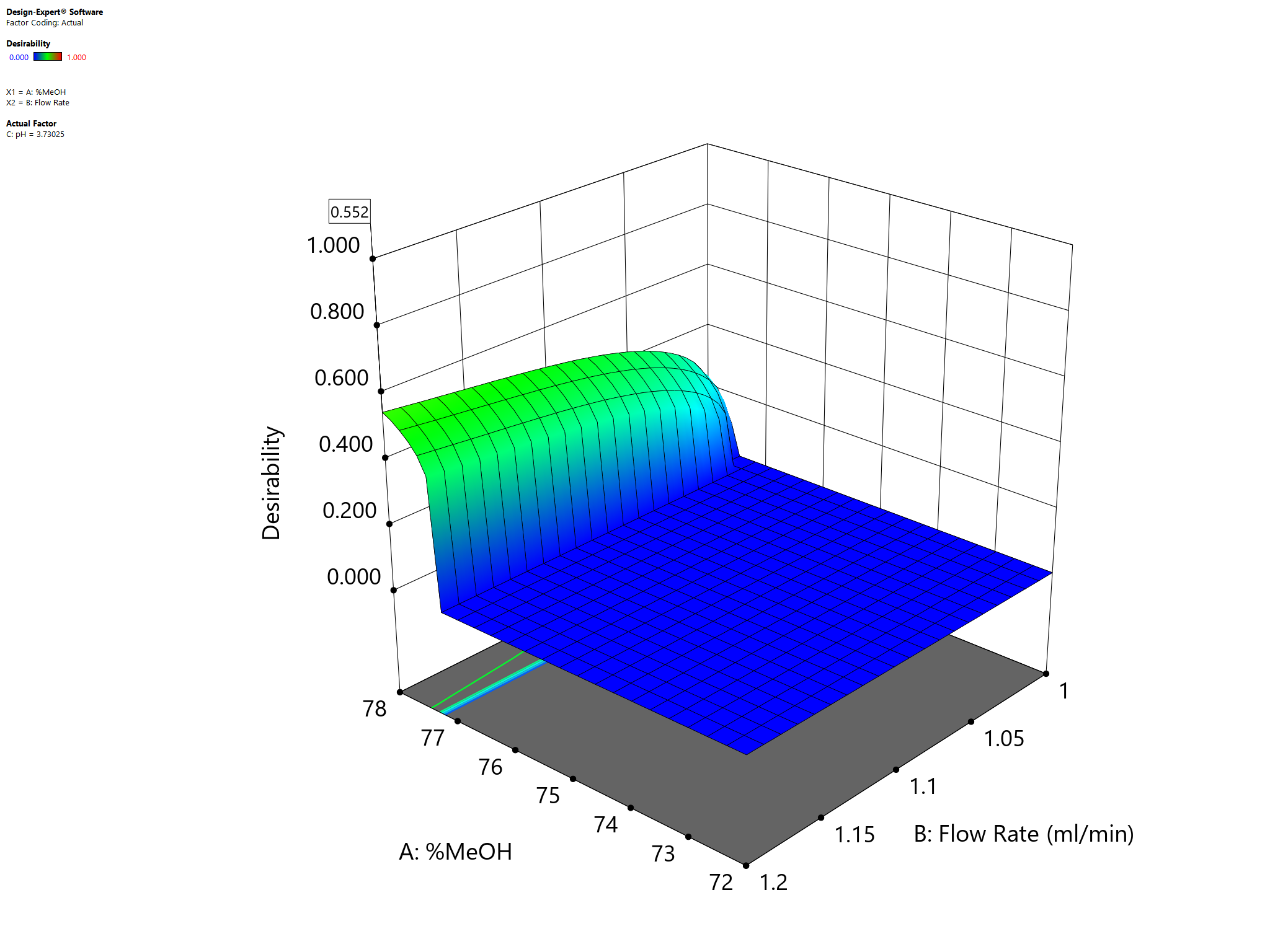

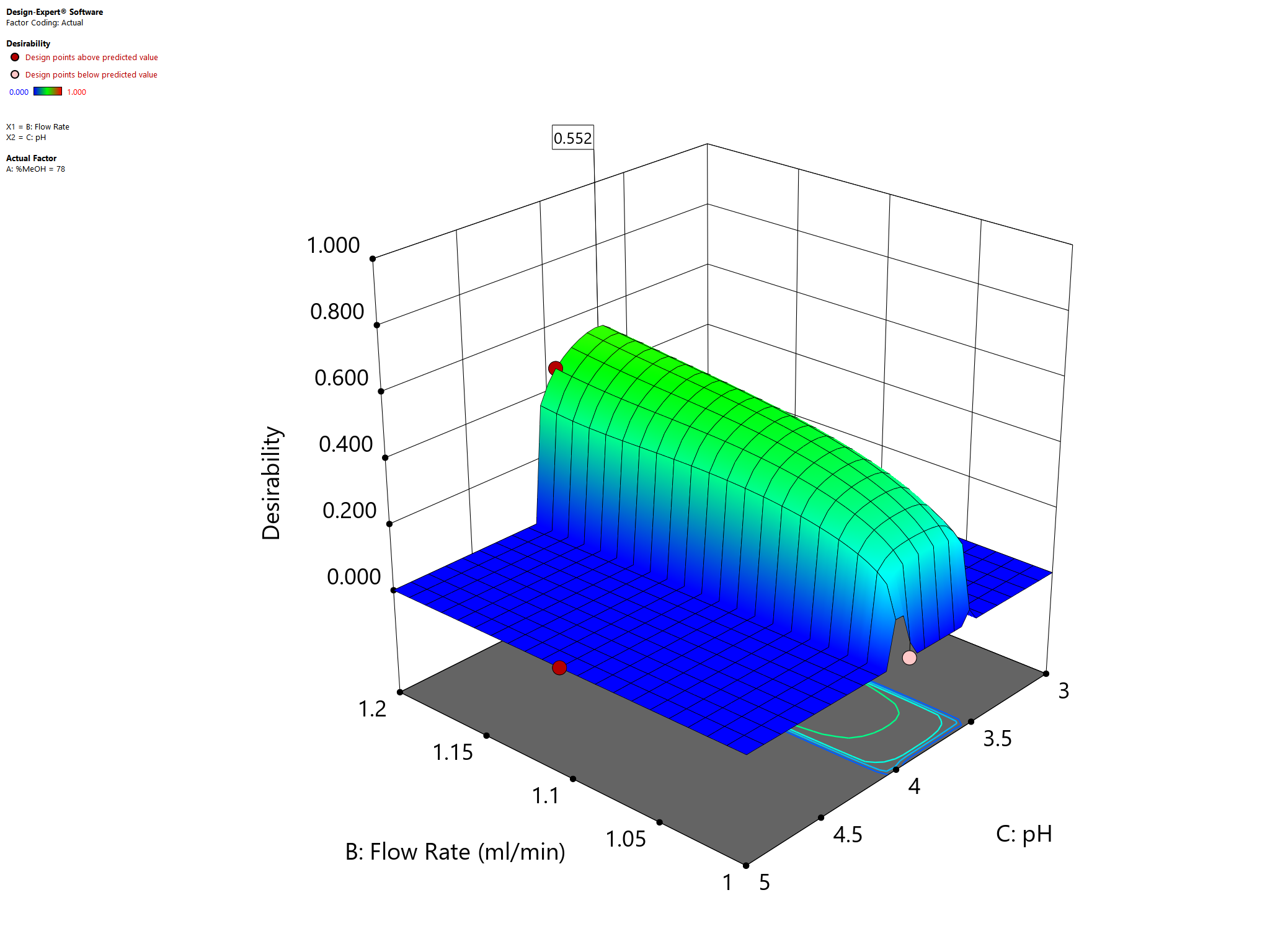

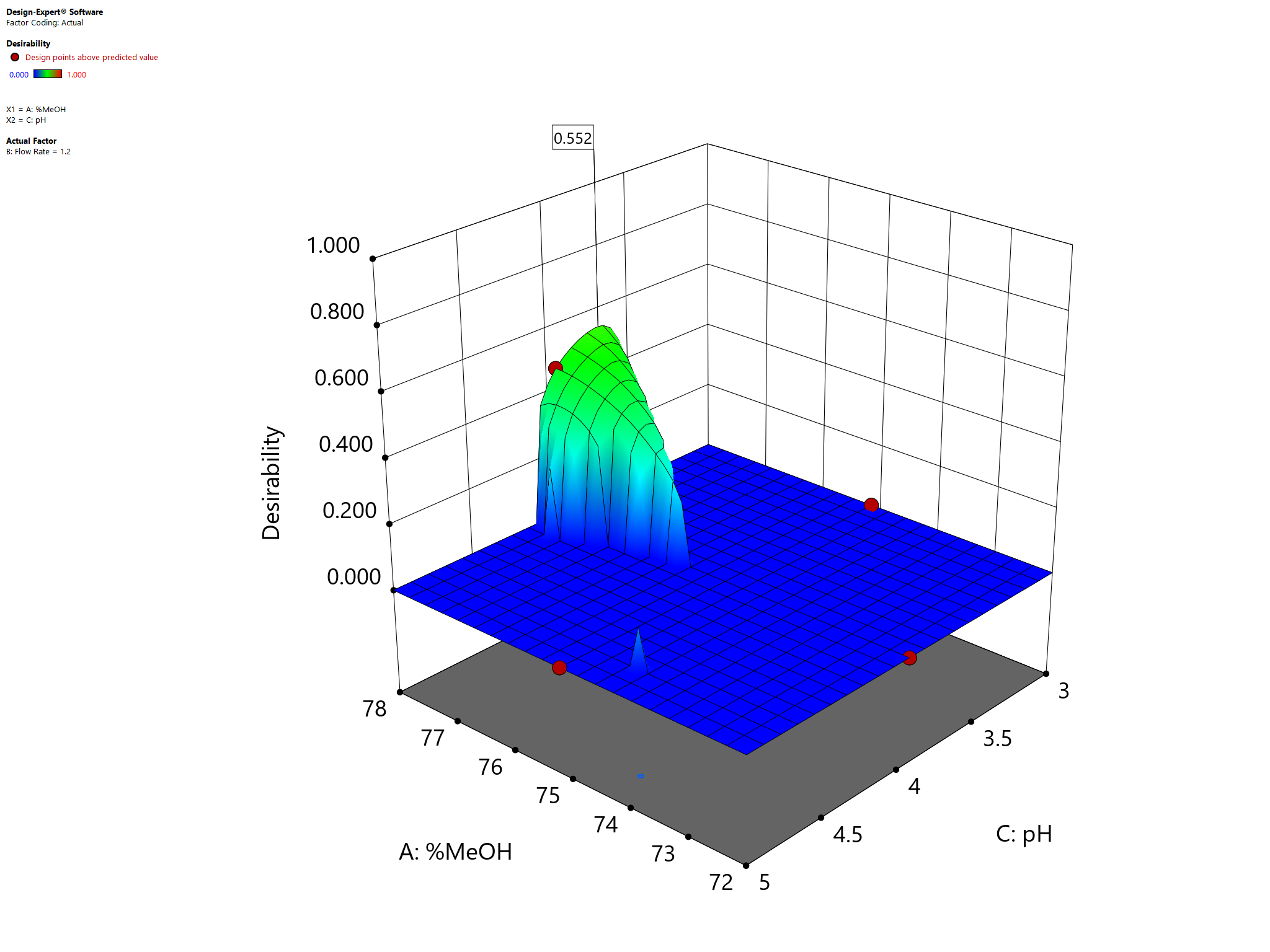


f

b

c


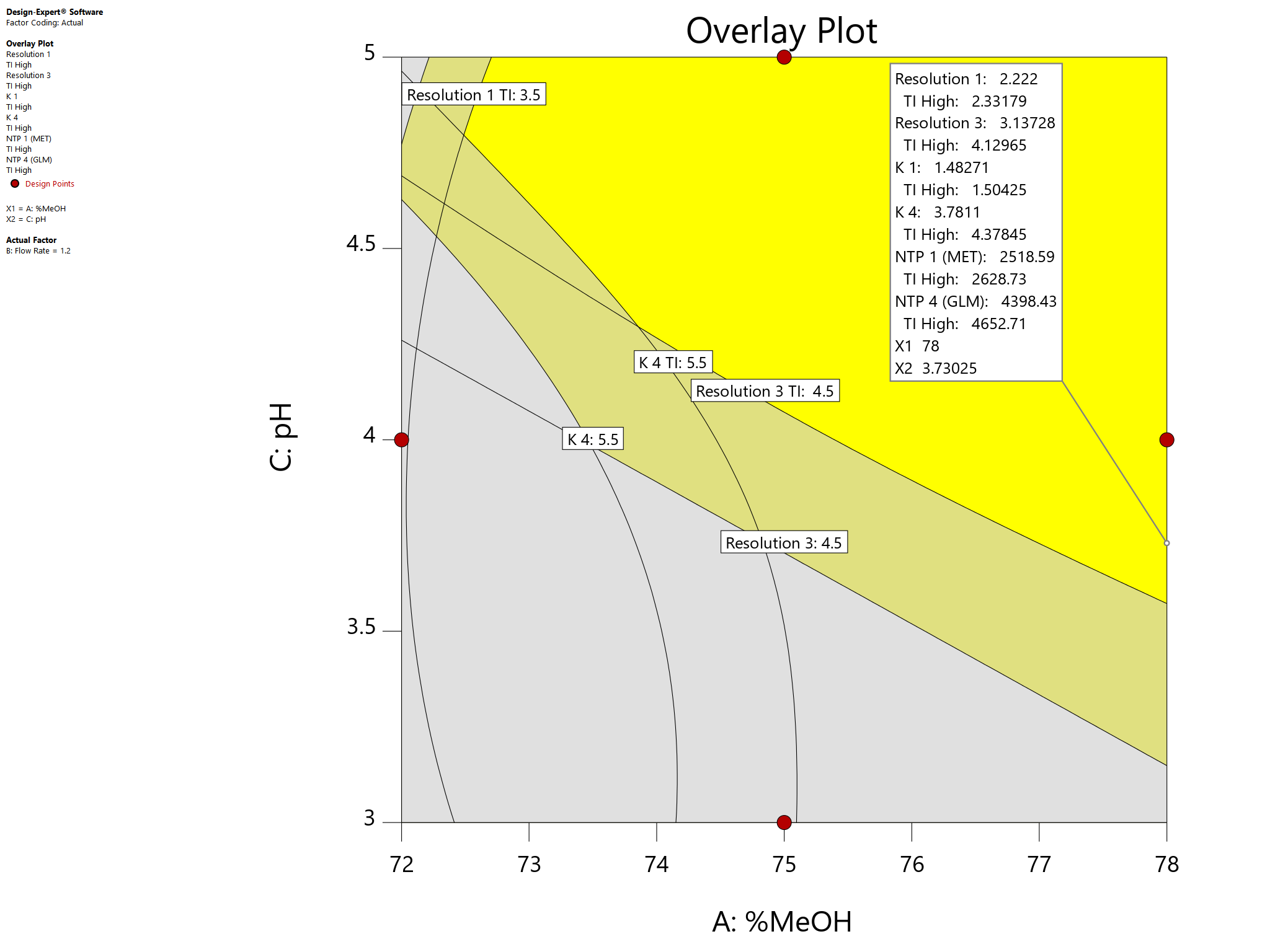

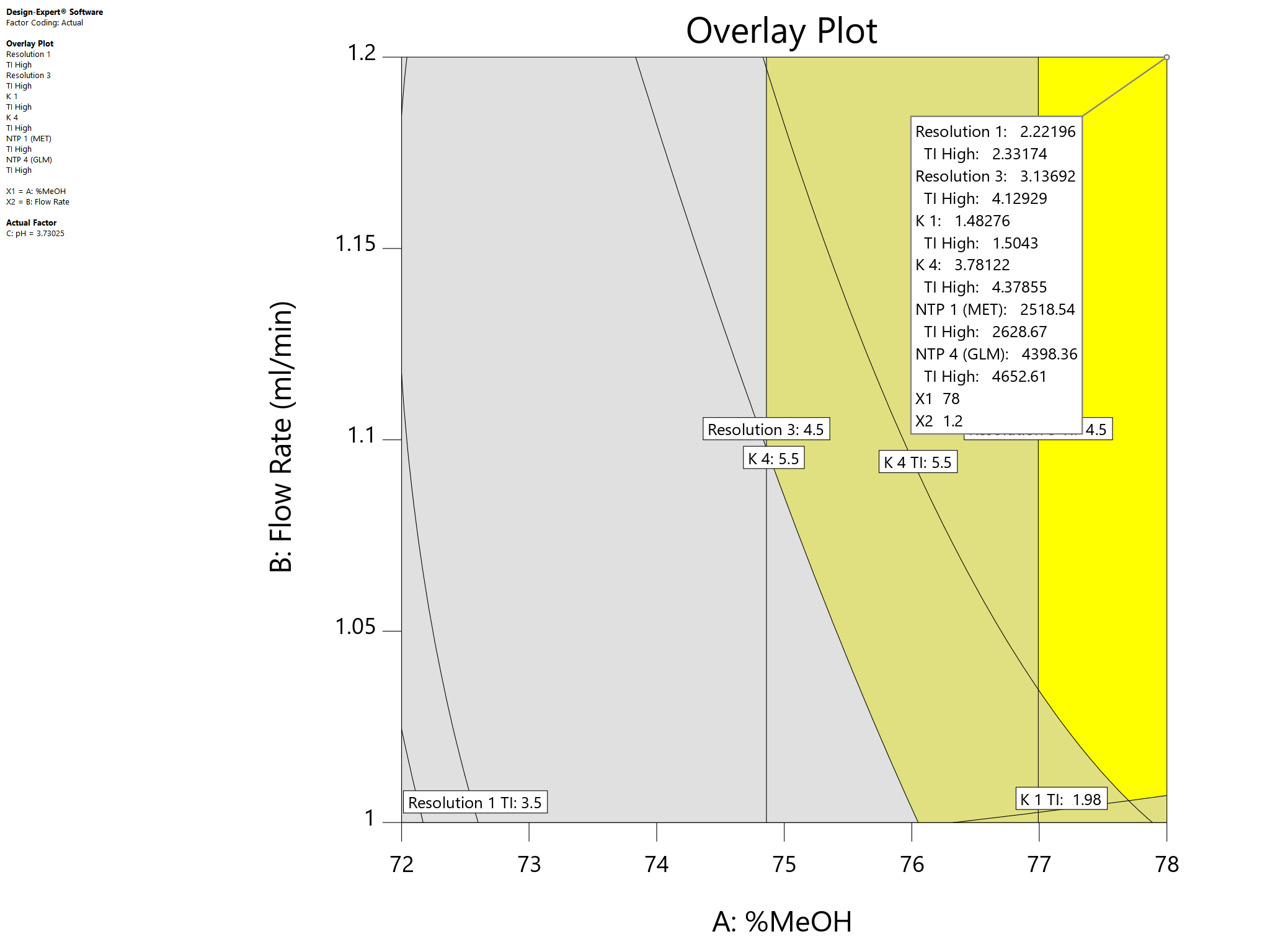

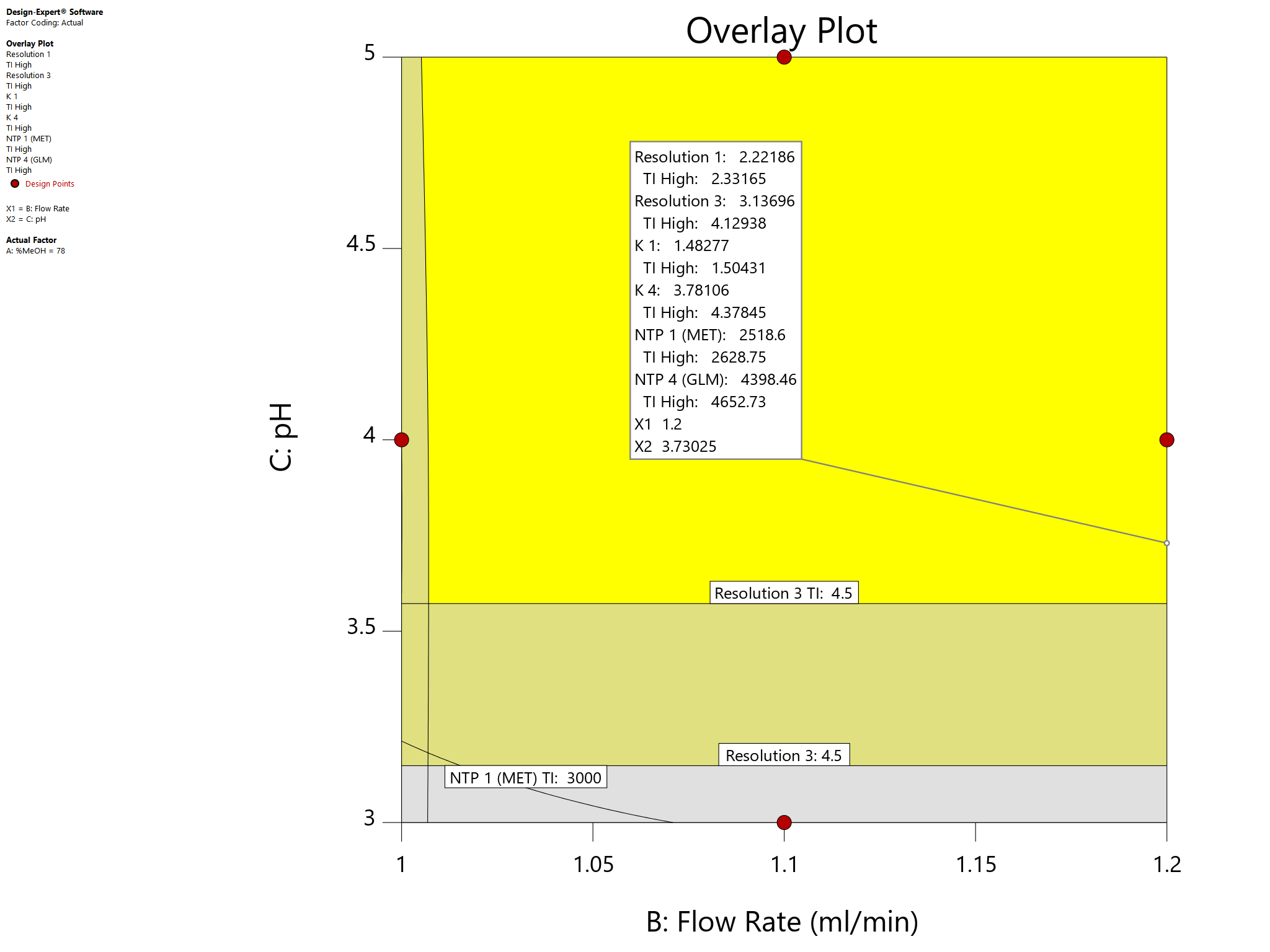


a

e

d

**Fig. S5:** Desirability and overlay plots


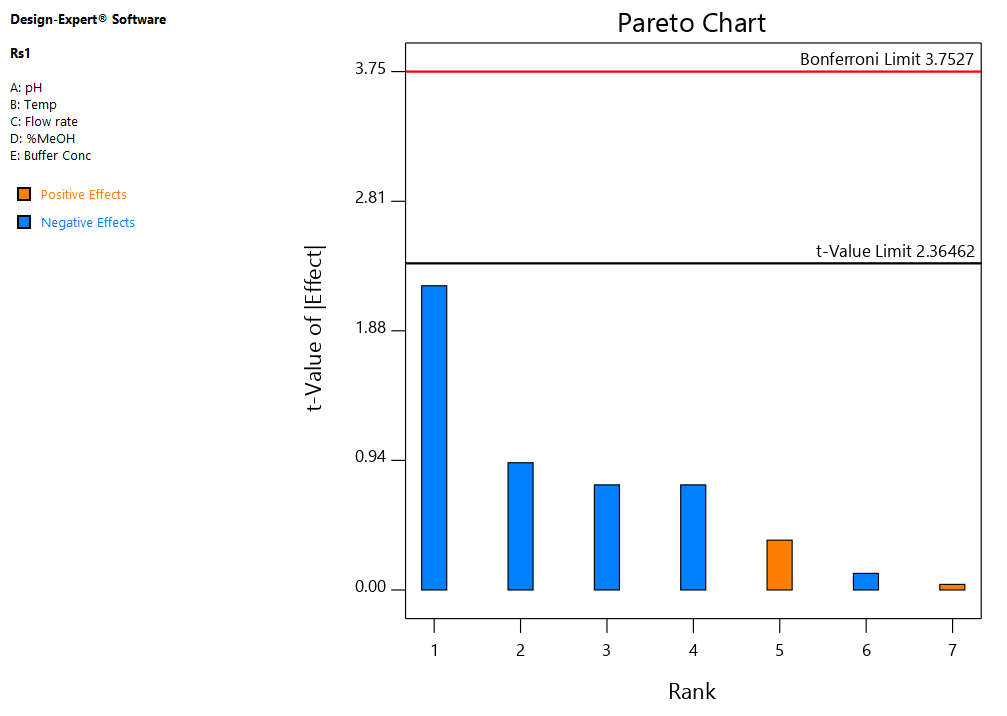

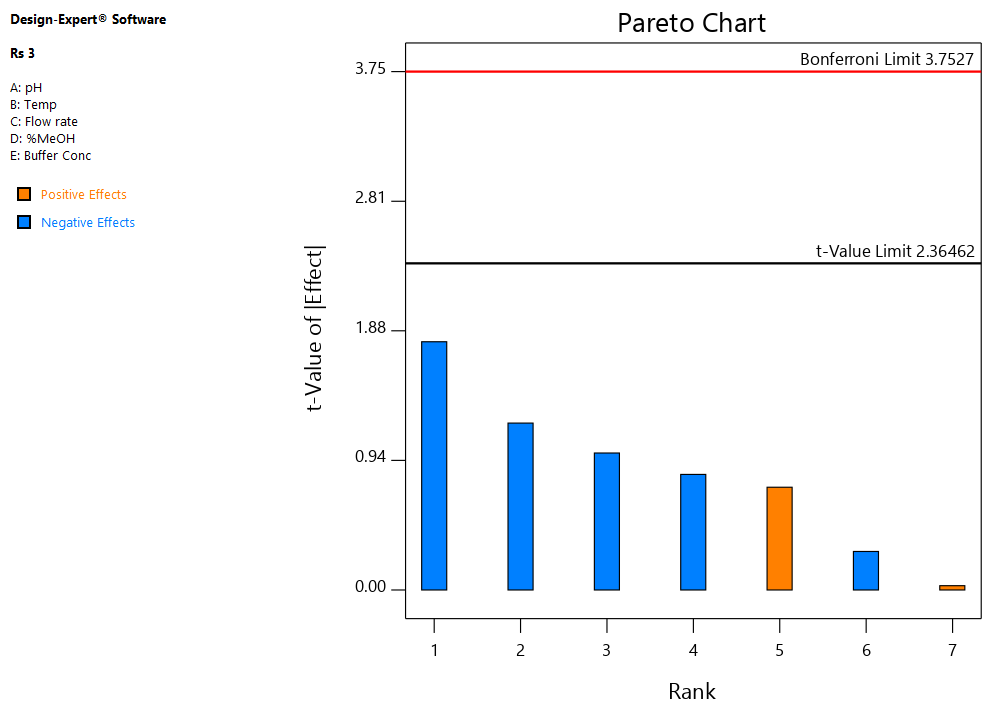

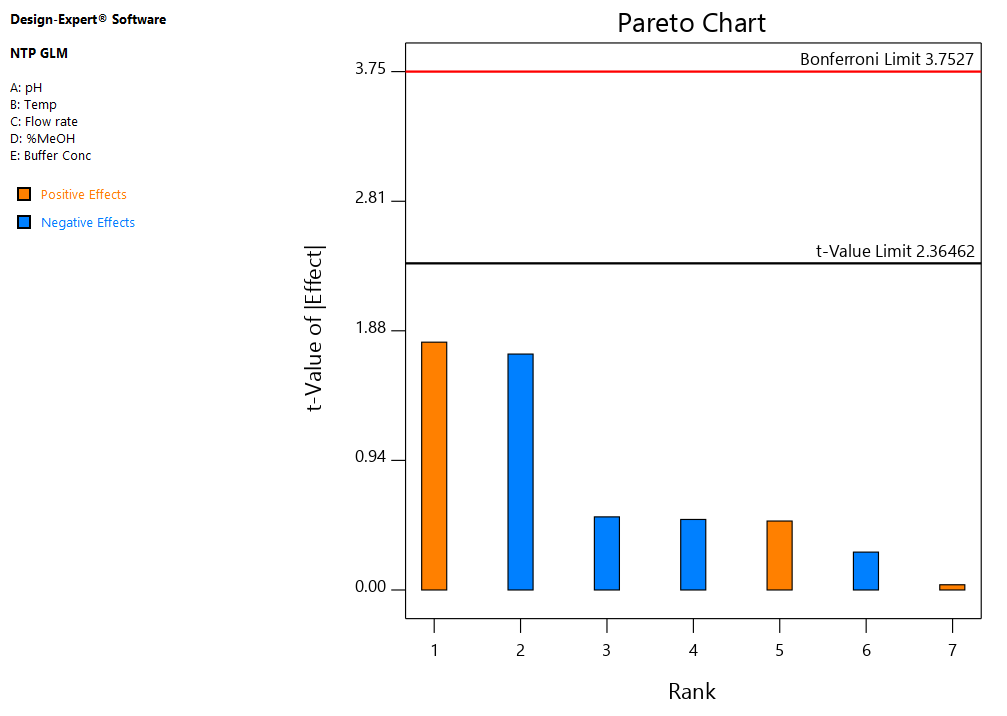

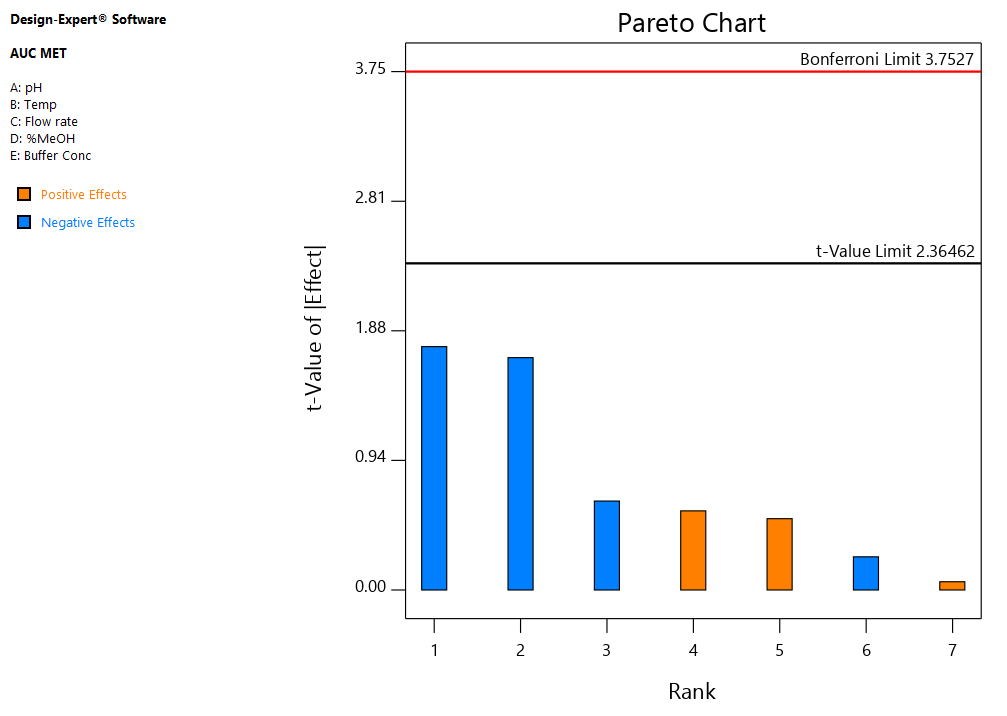

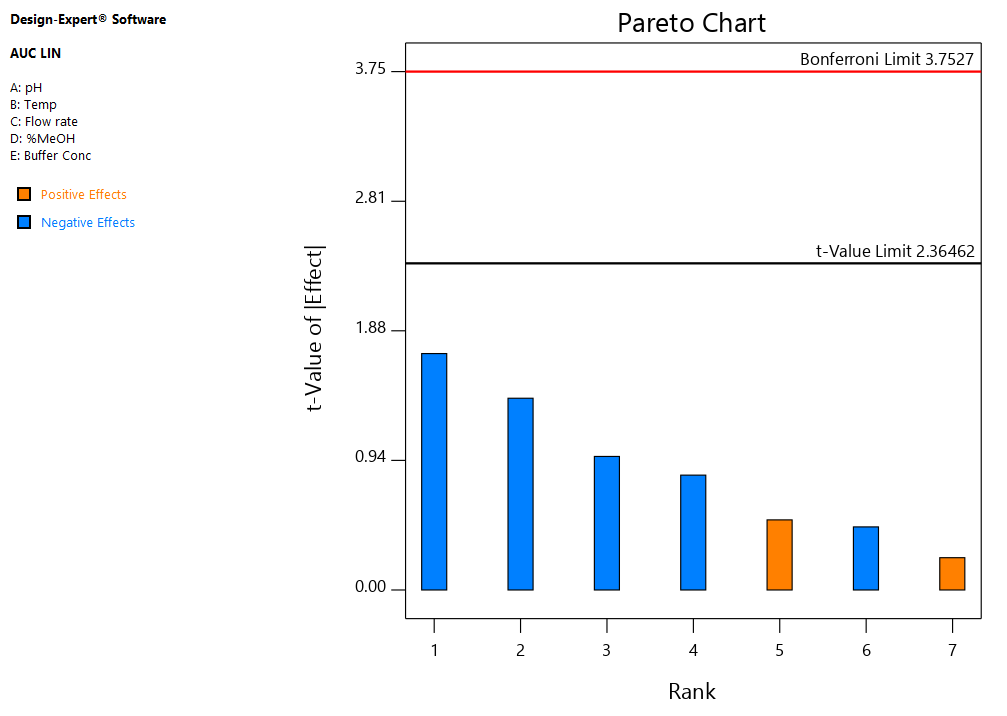

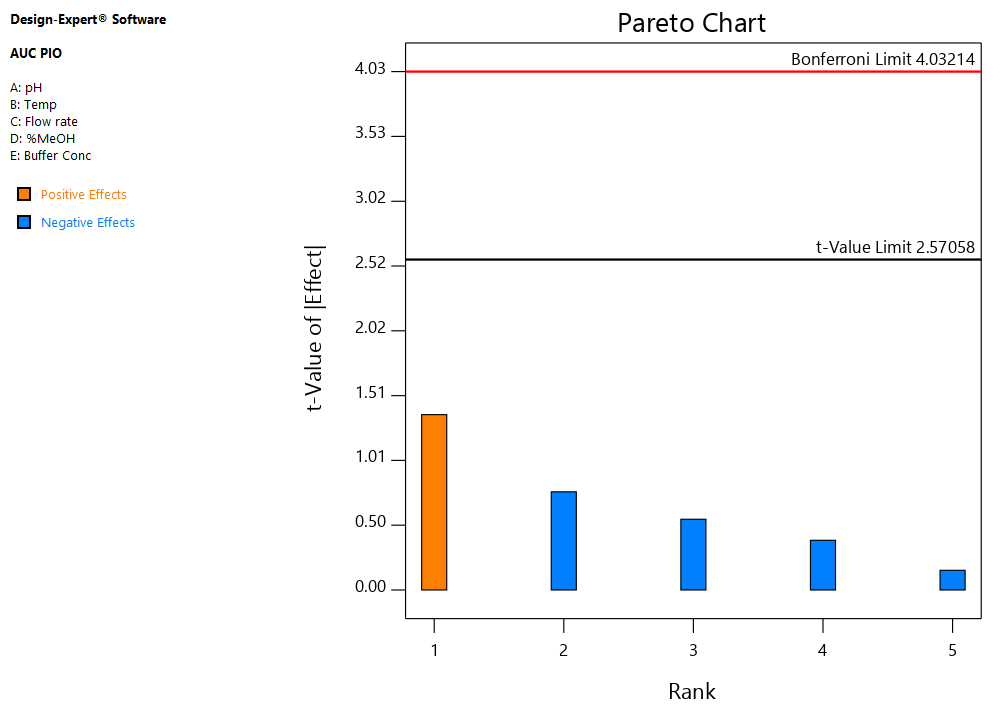

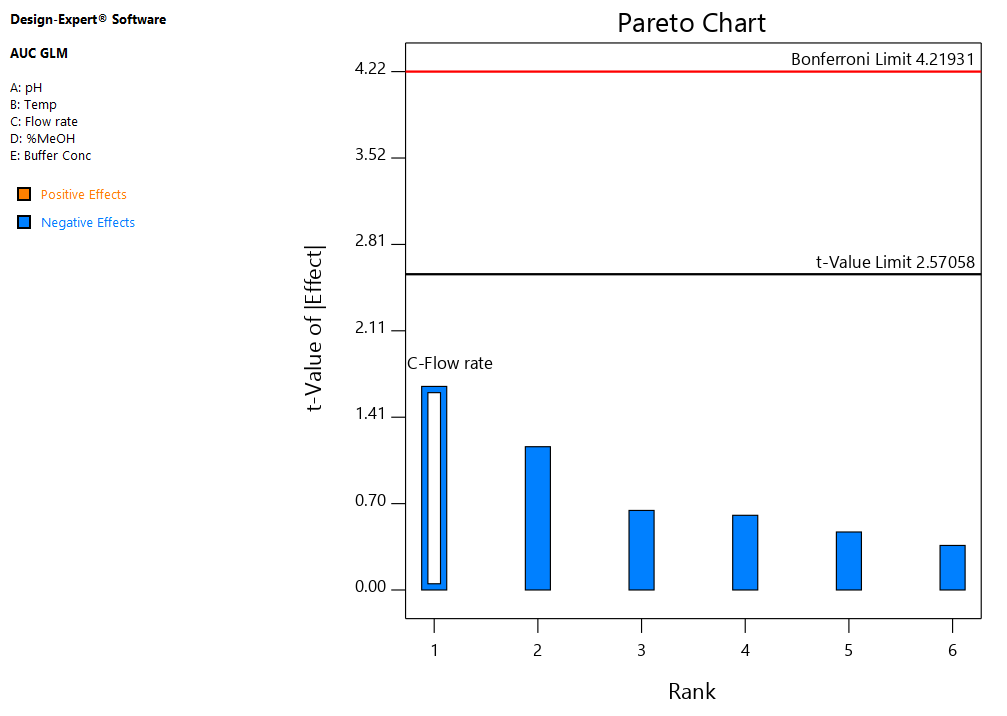


a

b

c

d

e

f

g

**Fig S6:** Pareto charts of robustness studies
